# High Efficacy of the Volatile Organic Compounds of *Streptomyces yanglinensis* 3-10 in Suppression of *Aspergillus* Contamination on Peanut Kernels

**DOI:** 10.3389/fmicb.2020.00142

**Published:** 2020-02-06

**Authors:** Ang Lyu, Long Yang, Mingde Wu, Jing Zhang, Guoqing Li

**Affiliations:** ^1^State Key Laboratory of Agricultural Microbiology, Huazhong Agricultural University, Wuhan, China; ^2^Key Laboratory of Plant Pathology of Hubei Province, Wuhan, China; ^3^School of Life Sciences and Technology, Hubei Engineering University, Xiaogan, China

**Keywords:** *Streptomyces yanglinensis* 3-10, volatile organic compounds, antifungal activity, *Aspergillus*, methyl 2-methylbutyrate, biofumigant

## Abstract

*Aspergillus flavus* and *Aspergillus parasiticus* are saprophytic fungi which can infect and contaminate preharvest and postharvest food/feed with production of aflatoxins (B_1_, B_2_, and G). They are also an opportunistic pathogen causing aspergillosis diseases of animals and humans. In this study, the volatile organic compounds (VOCs) from *Streptomyces yanglinensis* 3-10 were found to be able to inhibit mycelial growth, sporulation, conidial germination, and expression of aflatoxin biosynthesis genes in *A. flavus* and *A. parasiticus in vitro*. On peanut kernels, the VOCs can also reduce the disease severity and inhibit the aflatoxins production by *A. flavus* and *A. parasiticus* under the storage conditions. Scanning electron microscope (SEM) observation showed that high dosage of the VOCs can inhibit conidial germination and colonization by the two species of *Aspergillus* on peanut kernels. The VOCs also showed suppression of mycelial growth on 18 other plant pathogenic fungi and one Oomycetes organism. By using SPME-GC-MS, 19 major VOCs were detected, like in other *Streptomyces*, 2-MIB was found as the main volatile component among the detected VOCs. Three standard chemicals, including methyl 2-methylbutyrate (M2M), 2-phenylethanol (2-PE), and β-caryophyllene (β-CA), showed antifungal activity against *A. flavus* and *A. parasiticus*. Among them, M2M showed highest inhibitory effect than other two standard compounds against conidial germination of *A. flavus* and *A. parasiticus*. To date, this is the first record about the antifungal activity of M2M against *A. flavus* and *A. parasiticus*. The VOCs from *S. yanglinensis* 3-10 did not affect growth of peanut seedlings. In conclusion, our results indicate that *S. yanglinensis* 3-10 may has a potential to become a promising biofumigant in for control of *A. flavus* and *A. parasiticus*.

## Introduction

Volatile organic compounds (VOCs) are lipophilic chemicals with a low boiling point and low molecular mass (100–500 Da), but with high vapor pressure (Effmert et al., 2012). So far, there are approximately 1300 described VOCs from various microorganisms (Effmert et al., 2012; Lemfack et al., 2014; Piechulla and Degenhardt, 2014). The VOCs with antimicrobial activity have been reported in bacteria (Martins et al., 2019), filamentous fungi (Zhang et al., 2015), yeasts (Huang et al., 2011, 2012), algaes (Gong et al., 2015), and higher plants (Wood et al., 2013). The VOCs from *Streptomyces platensis* showed inhibitory activity against *Botrytis cinerea* on strawberry, *Rhizoctonia solani* on rice seedlings, and *Sclerotinia sclerotiorum* on oilseed rape (Wan et al., 2008). The VOCs from *Streptomyces globisporus* showed inhibitory activity against *Penicillium iltalicum* on citrus and *B*. *cinerea* on tomato (Li et al., 2010, 2012). The VOCs from *Streptomyces alboflavus* was able to inhibit the mycelial growth of several filamentous fungi (Wang et al., 2013). In addition, the VOCs produced by *Streptomyces* spp. also showed inhibition of mycelial growth (IMG) of *R. solani* and promote the growth of *Arabidopsis thaliana* (Cordovez et al., 2015). VOCs can diffuse to atmosphere and biodegradable which cannot cause the issue of toxic residues (Wang et al., 2013). Previous studies demonstrated that VOCs from endophytic fungus *Muscodor albus* and VOCs from *Saccharomyces cerevisiae* can be used as mycofumigant to control of many postharvest fruit diseases (Schnabel and Mercier, 2006; Toffano et al., 2017).

*Aspergillus* species are a threat to agriculture and human health, as some of them can produce carcinogenic and mutagenic secondary metabolites, like aflatoxin. Aflatoxin B_1_ is most frequently found in maize, peanut, and rice that shows the greatest toxigenic potential (Amaike and Keller, 2011). The International Agency for Research on Cancer (IARC) has classified AFB_1_ as a Group 1 human carcinogen (Williams et al., 2004). Aflatoxicosis is caused by inhaling or ingesting high levels of aflatoxin contaminated food and it is a major problem in developing countries, especially in Asia and Africa (Amaike and Keller, 2011). *Aspergillus flavus* and *Aspergillus parasiticus* may not reduce yield, but severe economic losses can be caused by the contamination of kernels or grains with aflatoxins produced by the fungi, especially under the storage conditions (Amaike and Keller, 2011). For instance, one major issue in peanut production worldwide is the aflatoxins contamination (Torres et al., 2014). China is the world’s largest producer of peanuts (USDA, 2013), while approximately 60% peanuts were contaminated by aflatoxins in six provinces of China (Gao et al., 2011). The most effective strategy to reduce and/or eliminate aflatoxins is to prevent aflatoxigenic *Aspergillus* spp. from colonization on food/feed products during storage (Gong et al., 2015).

To control *Aspergillus* spp. on peanuts, several chemicals and fungicides are applied to suppress the mycelial growth and aflatoxins production (Kolosova and Stroka, 2011). Additional concern about resistance and residue problems in application of chemicals compromises control efficacy of mycotoxin contamination indirectly (Torres et al., 2014). Biological control by applying competitive non-toxigenic isolates of *A. flavus* and/or *A. parasiticus* to soil has been explored in previous studies and achieved at least partial success, and a biocontrol product, namely, Aflasafe^TM^ was successfully commercialized (Atehnkeng et al., 2008). Zucchi et al. (2008) used cell suspension of *Streptomyces* sp. ASBV-1 to reduce aflatoxins accumulation by *A. parasiticus* on peanut. Reddy et al. (2010) reported the culture filtrate of *Rhodococcus erythropolis* completely inhibited the *A. flavus* growth and AFB_1_ production. Zhang et al. (2013) used antifungal substances purified from Streptomyces *hygroscopicus* to inhibit *A. flavus* on peanuts. Prasertsan and Sawai (2018) reported the VOCs from *Streptomyces mycarofaciens* showed antagonist to *A. flavus* and *A. parasiticus* growth on maize. Gong et al. (2015, 2019) reported that VOCs from *Shewanella algae* and *Alcaligenes faecalis* showed inhibitory activity against mycelial growth and aflatoxins production of *A. flavus*.

In this study, we found that the VOCs from *Streptomyces yanglinensis* 3-10 showed strong antifungal activity on mycelial growth and conidia germination, as well as on suppression of expression of aflatoxin biosynthesis genes in *A. flavus* and *A. parasiticus*. The VOCs can also reduce the contamination and aflatoxins produced by *A. flavus* and *A. parasiticus* on peanut kernels under storage condition. By using SPME-GC-MS, 19 major putative components of VOCs were identified and three pure chemicals were used in a bioassay to verify the antifungal activity against *A. flavus* and *A. parasiticus*. Methyl 2-methylbutyrate (M2M) and 2-phenylethanol (2-PE) were proved with inhibitory effect on conidial germination and mycelial growth of *Aspergillus* spp. We also found that the VOCs from *S. yanglinensis* 3-10 is not harmful to peanut seedling growth.

## Materials and Methods

### Microorganisms and Cultural Media

A total of 23 microbial isolates were used in this study, including isolate 3-10 of *S. yanglinensis* (Lyu et al., 2017), two isolates of *Pythium* species, and 20 isolates of fungi. Origin of these isolates was listed in Supplementary Table S1. Among these isolates, *S. yanglinensis* 3-10 was used to produce the VOCs. Two species of *Aspergillus*, namely, *A*. *flavus* NRRL3357 and *A*. *parasiticus* MO527, were used to infect peanut kernels, where they grew, sporulated, and produced aflatoxins B_1_, B_2_, G_1_, and G_2_ (AFB_1_, AFB_2_, AFG_1_, and AFG_2_, respectively). The remaining 20 isolates were used to determine the inhibitory spectrum of the VOCs of *S. yanglinensis* 3-10. Stock cultures of each isolate were maintained on potato dextrose agar (PDA) and stored in a refrigerator at 4°C. Working cultures were established on PDA by transferring mycelial agar plugs from a stock culture of each isolate and the cultures were incubated at 20°C in dark for 7–14 days.

Four cultural media were used in this study, including glucose agar (GA), ISP-2 liquid and agar media (ISP = International *Streptomyces* Project), PDA, and autoclaved wheat grains (AWG). GA contained (in 1000 mL water) 20 g D-glucose and 15 g agar, and it was used for determination of conidial germination of *A. flavus* and *A. parasiticus*. ISP-2 liquid medium contained (in 1000 mL water) 4 g D-glucose, 10 g malt extract, and 4 g yeast extract (pH 6.5–7.0) (Shirling and Gottlieb, 1966), and it was used for preparation of the seed cultures (inoculum) of *S. yanglinensis* 3-10. The ISP-2 agar medium was prepared by addition of agar (2%, w/v) in the ISP-2 liquid medium. Both the ISP-2 liquid medium and the ISP-2 agar medium were used for incubation of *S. yanglinensis* 3-10. PDA was prepared with peeled potato tubers purchased from a local supermarket using the procedure described by Fang (1998). The medium AWG was prepared with wheat grains using the procedure described by Zhang et al. (2015). Both potato tubers and wheat grains (cultivars unknown) were purchased from a local supermarket in Wuhan of China.

### Profiling of the *Streptomyces* VOCs

For preparation of the VOCs, an aliquot (1 mL) of a spore suspension (1 × 10^8^ spores per mL) of *S. yanglinensis* 3-10 was pipetted to a 250-mL Erlenmeyer flask containing 100 mL ISP-2 liquid medium. The flask was mounted on a rotary shaker and the culture was shake-incubated at 150 r/min at 28°C for 48 h. The resulting culture was used as the seed inoculum for inoculation of the AWG medium in 250-mL flasks each containing 80 g AWG. The ratio of the seed inoculum of *S. yanglinensis* 3-10 and AWG was 1:4 (v/w). The AWG cultures were incubated at 28°C in dark for 3, 7, 10, and 14 days for production of the VOCs.

For profiling of the VOCs, a flask with 80 g AWG culture of *S. yanglinensis* 3-10 (3, 7, 10, or 14-day-old) and another flask with 80 g fresh AWG (control) were maintained at 50°C for 30 min for emission of the VOCs. A fiber coated with VOC adsorbent, namely, divinyl-benzene/carboxen/polydimethylsiloxane (SUPELCO^®^, Bellfonte, PA, United States), was inserted into the airspace of a flask for 30 min to absorb the VOCs in that flask. The fiber was pulled out and immediately inserted into the injection port of a gas-chromatography and mass-spectrometry (GC-MS) instrument (Thermo Scientific DSQII, United States) equipped with an Agilent J & W HP-5MS fused-silica capillary column (30 m × 0.25 mm × 0.25 μm, length × inner diameter × film thickness) (Agilent Technologies Inc., Santa Clara, CA, United States). The GC-MS was performed using the procedures described in our previous study (Wan et al., 2008; Huang et al., 2011). Mass spectra were obtained using the scan modus with the total ion counts ranging from 45 to 650 *m/z*. The VOCs were identified by comparison of their mass spectra with those in the database of the National Institute of Standards and Technology (NIST)/EPA/NIH library (Version 2.0) deposited in GC-MS with the similarity index higher than 800. The VOCs detected both in the AWG cultures of *S. yanglinensis* 3-10 and in the fresh AWG were not considered to be the components produced by *S. yanglinensis* 3-10. The analysis was repeated once with three replicates both for the AWG cultures of *S. yanglinensis* 3-10 and for the fresh AWG.

### RT-PCR Detection of Expression of Selected VOC Synthase Genes in *S. yanglinensis*

Nineteen major VOCs were identified by GC-MS in the AWG cultures of *S. yanglinensis* 3-10 (Table 1 and Supplementary Figure S1). Production of five of the VOCs, including β-caryophyllene (β-CA), *trans*-1,10-dimethyl-*trans*-9-decalinol (geosmin), 2-methyl-2-bornene (2-M2B), 2-methylisoborneol (2-MIB), and 2-PE was confirmed by detection of expression of the genes responsible for biosynthesis of these VOCs in *S. yanglinensis* 3-10. First, the whole genome of *S. yanglinensis* 3-10 was sequenced by Novogene Co. Ltd. (Beijing, China). Then, the genome sequence of *S. yanglinensis* 3-10 was submitted to the AntiSMASH database^1^ and the KEGG pathway database^2^ for search of the VOC biosynthesis-related genes or pathways. Five genes coding for the VOC biosynthetic enzymes were found, including the genes coding for 2-MIB synthase (GenBank Acc. No. MK861971), methyltransferase (GenBank Acc. No. MK861972), geosmin synthase (GenBank Acc. No. MK861973), aryl-alcohol dehydrogenasae (GenBank Acc. No. MK861974), and (+)-β-CA synthase (GenBank Acc. No. MK861975) (Supplementary Figures S2–S14). The DNA sequences of these genes as well as the gene for DNA gyrase subunit B (gyrB) were used for designing specific oligonucleotide PCR primers. DNA gyrase subunit B (gyrB) gene was used as the reference gene. Total RNA was extracted using E.Z.N.A^®^ Bacterial RNA Kit (Omega Bio-tek, Inc., Norcross, GA, United States) from the mycelial masses of *S. yanglinensis* 3-10 harvested from the cultures (28°C) on ISP-2 agar medium. The extract was treated with DNase I (TaKaRa Biotechnol. Co. Ltd., Dalian, China) to eliminate DNA contamination. The RNA of ∼1 μg was reverse transcribed with the reagents in ThermoScript One Step RT-PCR Kit (TaKaRa Biomedical Technology Co., Ltd., Beijing, China). The resulting transcripts were then used as templates in PCR detection of the five VOC biosynthetic genes as well as the gyrB gene with the specific primers and the specific thermal programs (Supplementary Table S2). The PCR products were separated by agarose gel electrophoresis (1%, w/v) and the DNA bands were viewed on an UV trans-illuminator after staining with ethidium bromide solution (1.5 μg/L) for 10 min.

**TABLE 1:** GC-MS analysis of the major volatile organic compounds emitted by *Streptomyces yanglinensis* 3-10 in the autoclaved-wheat-grain (AWG) cultures after incubation at 28°C for 3, 7, 10, and 14 days.

Serial no.	RT (min)	Relative peak area (%)				Possible compound
		3 days	7 days	10 days	14 days	
1	3.4	2.1	2.4	1.0	0.6	Methyl 2-methylbutyrate (M2M)
2	9.2	0.8	1.2	2.1	0.9	2,6,6-Trimethyl-2,4-Cycloheptadien-1-one
3	10.5	15.7	12.4	11.9	8.6	2-Methyl-2-bornene (2-M2B)
4	11.1	1.0	1.5	0.9	1.6	Hexanoic acid, 2,4- dimethyl-, methyl ester, (2DL,4L)-
5	14.1	n.d.	2.8	3.1	4.3	3-Methyl-2-(2-methyl-2-butenyl)-furan
6	14.7	0.3	1.1	1.9	3.5	2-Phenylethanol (2-PE)
7	17.8	12.3	12.9	26.6	17.4	2-Methylisoborneol (2-MIB)
8	18.9	0.3	0.9	1.4	1.9	6-Camphenol
9	19.6	4.7	5.2	3.6	3.5	1H-Indene, 1-ethylideneoctahydro-7a- methyl-, (1Z, 3a, 7a-)
10	19.9	2.5	2.4	1.7	1.5	Cyclohexane,1,1,4,4-tetramethyl-2,6-bis (methylene)-
11	20.7	1.8	1.9	1.2	1.3	1H-Indene, 1-ethylideneoctahydro-7a- methyl-, cis-
12	27.2	2.5	4.1	3.8	3.6	*trans*-1,10-Dimethyl-*trans*-9-decalinol (geosmin)
13	28.8	0.8	0.8	0.6	0.6	(+)-β-Caryophyllene
14	29.5	1.9	2.4	1.9	2.5	Naphthalene, 1,2,3,5,6,7,8,8a-octahydro-1,8a-dimethyl-7-(1-methylethenyl)-, [1S-(1,7,8a]-
15	29.7	0.7	0.8	0.6	0.4	Copaene
16	29.8	1.0	1.2	0.6	0.7	(3R,5aS,9R,9aS)-2,2,5a,9-tetramethyloctahydro-2H-3,9a-methano-1-benzoxepine
17	30.4	2.4	2.7	1.8	1.4	Muurolene
18	30.5	1.7	1.8	1.5	1.4	(S,1Z,6Z)-8-Isopropyl-1-methyl-5-methylenecyclodeca-1,6-diene
19	32.3	3.4	2.9	1.6	2.3	Naphthalene,1,2,3,5,6,8a-hexahydro-4,7-dimethyl-1-(1-methylethyl)-, (1S-cis)-

*RT = retention time; n.d. = not detectable.*

### Antifungal Activity of the Selected VOCs Against *Aspergillus*

Three synthetic chemicals present in the VOC profile of *S. yanglinensis* 3-10, including 2-PE, M2M, and β-CA, were selected for testing their antifungal activity against *A. flavus* and *A. parasiticus.* The chemicals (purity: > 98.5%) were purchased from Sigma–Aldrich^®^ Company (St. Louis, MO, United States). IMG and conidial germination by these chemicals was determined in two-compartment plastic Petri dishes (9 cm in diameter). In the bioassay for IMG, 10 mL melted PDA was poured into one compartment of a dish. A mycelial agar plug (6 mm in diameter) of *A. flavus* or *A. parasiticus* from the margin area of 3-day-old PDA cultures (28°C) was placed on PDA in that compartment. Then, two filter paper pieces (FPPs) of approximately 1.6 × 1.5 cm (length × width) in size were placed in the other compartment of that dish. A synthetic chemical was pipetted to the two FPPs at 2.5, 5.0, 12.5, 25.0, 50.0, or 100 μL on each FPP. In the dish for the control treatment (CK), sterile distilled water was added to the two FPPs, 50 μL on each FPP. There were four dishes as four replicates for each chemical at each dosage and the control treatment. The dishes were individually sealed with parafilm (Laboratory Parafilm^®^ “M,” Neenah, WI, United States), and placed in an incubator at 28°C in dark for 3 days. Diameter of the colony of *A. flavus* or *A. parasiticus* in each dish was measured and percentage of IMG was calculated using the following formula:

IMG(%)=(AD-CKD)VOC/(AD-CK6)×100

where AD_CK_ represents the average colony diameter of *A. flavus* or *A. parasiticus* in the control treatment; D_VOC_ represents colony diameter of *A. flavus* or *A. parasiticus* in a dish for the treatment of an investigated VOC chemical at a given dosage; the value “6” represents diameter of the mycelial agar plug of *A. flavus* or *A. parasiticus*. The concentration for 50% IMG (IC_50_ in μL/mL) by a chemical was inferred based on the data about IMG and the dosages of that VOC chemical applied to the dishes (Huang et al., 2011).

In the bioassay for inhibition of conidial germination, 10 mL of melted GA medium was poured into a compartment of a two-compartment dish. An aliquot (100 μL) of the conidial suspension (1 × 10^6^ conidia/mL) of *A. flavus* or *A. parasiticus* harvested from 3-day-old PDA cultures (28°C) were pipetted onto that compartment, and the conidial suspension drop was evenly spread using a sterilized glass spatula. Meanwhile, a synthetic chemical was pipetted to two FPPs in the other compartment of that dish at 2.5, 5.0, 12.5, 25.0, 50.0, or 100 μL on each FPP. In the dish for the control treatment, conidia of *A. flavus* or *A. parasiticus* were plated on GA in one compartment and sterile distilled water was added to the two FPPs in the other compartment, 50 μL on each FPP. There were four dishes as four replicates for each VOC chemical at each dosage and for the control treatment. The dishes were sealed with the parafilm and placed in an incubator at 28°C for 12 h. Conidial germination on GA in each dish was observed under a compound light microscope by randomly counting at least 100 conidia in each dish. Then, percentage of germinated conidia was calculated. A conidium was considered to have germinated when the length of the germ-tube was equal to or longer than the diameter of that conidium. The percentage of inhibition to conidial germination of *A. flavus* or *A. parasiticus* by a VOC chemical was calculated based on difference in percentages of the germinated conidia between the control treatment and the treatment with that VOC chemical at a given dosage. The IC_50_ value for that VOC chemical was thus inferred based on the data about percentages of inhibition to conidial germination and the dosage of the chemical applied to the dishes (Huang et al., 2011).

### Suppression of Mycelial Growth and Sporulation of *Aspergillus* by the VOCs of *S. yanglinensis* 3-10

Two bioassays were carried out to determine the efficacy of the VOCs of *S. yanglinensis* 3-10 in suppression of mycelial growth and sporulation by *A. flavus* and *A. parasiticus*. The first bioassay is a time-course trial, aiming at determination of the time-course of production of the VOCs by *S. yanglinensis* 3-10 in AWG. *S. yanglinensis* 3-10 was inoculated in flasks containing AWG (80 g per flask) and the cultures were incubated at 28°C in dark for 3, 7, 10 and 14 days. They were used as source of the VOCs in determination of antifungal activity against *A. flavus* and *A. parasiticus* in double-dish sets (DDSs) described by Huang et al. (2011). A DDS consisted of two cover-free bottom glass dishes (9 cm in diameter), one dish was loaded with 10 g AWG culture of *S. yanglinensis* 3-10 at a given incubation time and the other bottom dish with PDA (20 mL) was inoculated in the center with a mycelial agar plug (6 mm in diameter) of *A. flavus* or *A. parasiticus*. For the control treatment (CK), one bottom dish was loaded with 10 g fresh AWG and the other bottom dish with PDA (20 mL) was inoculated with *A. flavus* or *A. parasiticus*. The two bottom dishes for each treatment were put together in an opposite direction (upper dish with *A. flavus*/*A. parasiticus*, lower dish with *S. yanglinensis*/fresh AWG) and sealed with a piece of parafilm (Huang et al., 2011). There were three DDSs as three replicates for each treatment. The DDSs were placed at 28°C in the dark for 3 days. Colony diameter of *A. flavus* or *A. parasiticus* in each DDS was measured. Meanwhile, the conidia of *A. flavus* or *A. parasiticus* in the PDA dish of each DDS were washed off using 20 mL water amended with 0.1% Tween 20 (v/v). The concentration of the conidia in the resulting conidial suspension was determined with the aid of a hemocytometer under a compound light microscope. Conidial yield (conidia/mm^2^) in each culture was calculated with the data on total conidial number and colony size of that culture.

The second bioassay is a dosage trial, aiming at determination of the antifungal activity of the VOCs from different dosages of the 7-day-old AWG cultures of *S. yanglinensis* 3-10 (VOCs^3–10AWG^). A DDS was established with a bottom dish containing 40 g fresh AWG (control), or the AWG culture of *S. yanglinensis* 3-10 at 5, 10, 20, 30, or 40 g per dish, and another bottom dish containing PDA inoculated with a mycelial agar plug of *A. flavus* or *A. parasiticus*. The DDSs were individually sealed with parafilm. There were three DDSs as three replicates for VOCs^3–10AWG^ of each dosage and the control treatment. The DDSs were placed at 28°C in dark for three days. Colony diameter and number of conidial yield (conidia/mm^2^) were measured using the procedures described above.

Additionally, the VOCs^3–10AWG^ were determined for suppression of mycelial growth of 20 other fungi and two species of *Pythium* (Supplementary Table S1) in DDSs using the procedures described above. A DDS in the treatment of the VOCs^3–10AWG^ was established with a bottom dish with PDA, which was inoculated with a target organism, and another bottom dish, which was loaded with 10 g 7-day-old AWG culture of *S. yanglinensis* 3-10. In the control treatment, a DDS consisted of a bottom dish with PDA, which was also inoculated with the same target organism, and another bottom dish with 10 g fresh AWG. For each target organism, there were three DDSs as three replicates for VOCs^3–10AWG^, and other three DDSs as three replicates for the control treatment. The DDSs were incubated at 20, 25, or 28°C for 1–7 days depending on thermal adaptation of the target organism. Diameter of the colony in each DDS was measured. The IMG value against each target organism was calculated using the formula mentioned above.

### Suppression of Conidial Germination of *Aspergillus* by the VOCs of *S. yanglinensis* 3-10

Both *A. flavus* and *A. parasiticus* were inoculated on PDA and the cultures were incubated at 28°C for 3 days. Conidia of each fungus were harvested from the PDA cultures by washing with sterile distilled water. The mixtures with conidia and hyphal fragments were filtered through four-layered cheesecloth to remove the hyphal fragments. The conidial concentration in the resulting conidial suspension was adjusted to 1 × 10^6^ conidia/mL with sterile distilled water. Aliquots of the conidial suspension of *A. flavus* or *A. parasiticus* were pipetted onto the GA medium in Petri dishes (9 cm diameter) at 200 μL per dish and the conidia in the conidial suspension drop were evenly spread using a sterilized glass spatula. There were two bioassays in this experiment, the time-course bioassay and the dosage bioassay. In the time-course bioassay, a DDS was established with two bottom dishes, one bottom dish containing GA was inoculated with the conidia of *A. flavus* or *A. parasticus*, and another bottom dish was loaded with 10 g fresh AWG (CK) or 10 g AWG cultures of *S. yanglinensis* 3-10 of a given incubation time (3-, 7-, 10-, or 14-day old). There were three DDSs as three replicates for each treatment and the DDS cultures were then incubated at 28°C in dark for 12 h. Conidial germination of *A. flavus* or *A. parasiticus* on GA in each DDS was observed under microscope by randomly counting at least 100 conidia on GA. Meanwhile, length of at least 50 randomly selected germ tubes in that DDS was measured.

In the dosage bioassay, a DDS was established with a bottom dish containing the conidia of *A. flavus A. parasticus* on GA, and another bottom dish containing 40 g fresh AWG (CK) or the 7-day-old AWG culture of *S. yanglinensis* 3-10 of a given dosage (5, 10, 20, 30, or 40 g per dish). There were three DDSs for each treatment as three replicates. The DDSs were individually sealed with parafilm and placed at 28°C in dark for 12 h. Conidial germination of *A. flavus* or *A. parasiticus* in each DDS was observed and length of germ tubes of each fungus was measured.

### *Streptomyces* VOC-Mediated Suppression of *Aspergillus* Infection of Peanut Kernels

Kernels of peanut (*Arachis hypogaea* L., cultivar unknown) were purchased from a local supermarket in Wuhan of China. They were soaked in sterile distilled water for 4 h, followed by surface sterilization in 70% ethanol (v/v) for 2 min and rinsing in sterile distilled water for three times, 1 min each time. Then, the kernels were blotted dry on pieces of sterilized paper towels and loaded in Petri dishes (6 cm in diameter), 10 kernels per dish. The cover of the dishes was removed and the bottom dishes with the peanuts were placed in a laminar flow hood for 30 min for evaporation of the water remains on the peanut kernel surface. Meanwhile, conidia of *A. flavus* and *A. parasiticus* were harvested from the PDA cultures (28°C, 3 days) by washing with sterile distilled water. The resulting conidial suspensions (1 × 10^6^ conidia/mL) were amended with 0.5% D-glucose (w/v), which served as the exogenous nutrient for triggering germination of the conidia. For each tested fungus, aliquots of the conidial suspension were pipetted to the peanut kernels in the dishes, 500 μL per dish and 42 dishes for each fungus. The dishes were gently shaken to ensure that all the kernels were contaminated with the conidia. The kernel-containing dishes inoculated with each fungus were divided into six lots as six treatments (seven dishes in each lot), one control treatment with VOCs from the fresh AWG (VOCs^AWG^) and five treatments with the VOCs from *S. yanglinensis* (VOCs^3–10AWG^).

The bioassay was done in 12 glass desiccators (∼5.8 L in airspace), six for *A. flavus* and another six for *A. parasiticus*. For each fungus, the desiccators for the control treatment was loaded at the bottom with 500 g fresh AWG as source of VOCs^AWG^, a seven-dish lot with the *A. flavus*- or *A. parasiticus*-inoculated peanut kernels were placed on the perforated ceramic clapboard (Supplementary Figure S15). Five other desiccators were loaded at the bottom with the 7-day-old AWG cultures of *S. yanglinensis* 3-10 at 100, 200, 300, 400, and 500 g per desiccator (equivalent to 17, 34, 52, 69, and 86 g/L, respectively). Five other six-dish lots with the *A. flavus-* or *A. parasiticus-*inoculated kernels were placed on the perforated ceramic clapboards of those desiccators, seven dishes in each desiccator. These five treatments were designated as VOCs^3–10AWG^-17, VOCs^3–10AWG^-34, VOCs^3–10AWG^-52, VOCs^3–10AWG^-69, and VOCs^3–10AWG^-86. The desiccators were covered with the lids, sealed with parafilm, and finally maintained in an incubator at 28°C in dark for 7 days. The kernels in three of the seven dishes in a desiccator (for a treatment) were individually rated for disease severity using a numerical scale of 0–5, where 0, healthy without visible mycelia or sporulation on the kernel surface; 1, sparse mycelia on the kernel surface without visible sporulation; 2, dense mycelia on the kernel surface without visible sporulation; 3, dense mycelia on the kernel surface with sparse sporulation; 4, dense mycelia on the kernel surface with moderate sporulation; and 5, dense mycelia on the kernel surface with vigorous sporulation. Then, the kernels in each dish were transferred to a 250-mL flask containing 50 mL water amended with 0.1% Tween 20 (v/v). The flask was stirred for 5 min to wash the conidia off. The mixture was filtered with four layers of cheesecloth to obtain the conidial suspension, which was consequently determined for conidial concentration using a hemocytometer. The conidial yield per kernel was calculated based on the data about conidial concentration, volume of the conidial suspension, and number of the kernels. The kernels in four other dishes in that desiccator were used for scanning electron microscope (SEM) observation of fungal colonization and sporulation and quantification of the content of aflatoxins with the following procedures.

### Scanning Electron Microscopy

A peanut kernel from one of the seven dishes in a desiccator (for a treatment) was randomly selected for SEM observation of colonization and sporulation of the two fungi on the kernel surface. The peel of each kernel was carefully taken off and cut into to small pieces (∼3 × 3 mm, length × width) using a sharp razor blade. The kernel peel pieces were immediately fixed in the glutaraldehyde fixative, followed by dehydration with gradient ethanol, drying in a Critical Point Dryer (Model: 13200E-AB, SPI SUPPLIES, West Chester, PA, United States), and gold-coating in a sputter coater (Model: JFC-1600, NTC, Tokyo, Japan) using the conventional procedures. Finally, the specimens were observed under a SEM (Model: JSM-6390/LV, NTC, Tokyo, Japan).

### Quantification of the Aflatoxins in Peanut Kernels

The kernels in three of the seven dishes in a desiccator (for a treatment) were dried at 50°C for 3 days and ground to fine powder using a mortar and pestle. The powder (5 g) for each treatment was suspended in 25 mL 70% methanol (v/v) in a 50-mL plastic tube, followed by sonication for 60 min and centrifugation at 5000 × *g* for 10 min to remove the granules in the suspension. The resulting supernatant was transferred to a new plastic tube and hexane was added at the volume ratio of 1:1 to extract aflatoxins (Gong et al., 2015). The upper hexane layer (500 μL) was pipetted out and used for identification and quantification of the aflatoxins by LC-MS (Waters ACQUITY UPLC H-Class system coupled to the XEVO TQ-S tandem quadrupole, Waters Cooperation, Milford, MA, United States). The mobile phase for the linear gradient washing consisted of two components, namely, A (MeOH) and B (5 mmol/L ammonium acetate, 0.05% formic acid in water). The washing lasted for 7 min with the program being set as follows: 1 min with A + B (20% + 80%); 3 min also with A + B (A: 20%→100%, B: 80%→0%); 1 min with A alone; 0.5 min with A + B (A: 100%→20%, B: 0%→80%); and 1.5 min also with A + B (A: 20%, B: 80%). The flow rate was adjusted to 0.3 mL/min. AFB_1_, AFB_2_, AFG_1_, and AFG_2_ were identified based on the molecular ion peaks (*m/z*) at 313, 315, 329, and 331, respectively (Nonaka et al., 2009). The standard AFB_1_, AFB_2_, AFG_1_, and AFG_2_ (Sigma–Aldrich^®^, St. Louis, MO, United States) were used as reference in identification and quantification.

### Determination of Expression of the Aflatoxins Biosthynesis Genes

The conidia of *A. flavus* or *A. parasiticus* were harvested from 3-day-old PDA cultures and then spread on a cellophane film placed on PDA in a Petri dish (9 cm in diameter) with 200 μL conidial suspension (1 × 10^7^ conidia/mL). Another Petri dish was loaded with 10 g the 7-day-old AWG culture of strain 3-10 or 10 g fresh AWG (CK). Then, the two dishes were face-to-face sealed by parafilm to form a DDS. After co-culturing at 28°C for 72 h, the mycelia on the film were collected and immediately frozen in liquid nitrogen. Total RNA in the mycelial sample was extracted using E.Z.N.A^®^ Fungal RNA Kit (Omega Bio-tek, Inc., Norcross, GA, United States) according to the provided manual. Expression of eleven important genes (*aflR*, *AccC*, *aflCa*, *aflA*, *aflS*, *aflO*, *aflD*, *aflF*, *aflP*, *aflQ*, *and aflX*) in the aflatoxins biosynthesis pathway in *A. flavus* and *A. parasiticus* were determined by quantitative real-time PCR (qRT-PCR) using the method described by Gong et al. (2019). The primers used for the qRT-PCR are listed in Supplementary Table S3.

### Effect of the VOCs of *S. yanglinensis* on Growth of Peanut

This is a VOCs-fumigation bioassay, aiming at determining the effect of the VOCs from *S. yanglinensis* 3-10 on growth of peanut seedlings. Peanut kernels (*A. hypogaea* cultivar: Zhonghua No. 12) were soaked in water for 12 h and placed on moisturized filter papers in Petri dishes (15 cm in diameter), 30 kernels per dish. The dishes were maintained at 28°C under the lighting regime of 12-h light and 12-h dark for 3 days. The pre-germinated peanut kernels were sown in plant culture mix in plastic pots (9.5 cm × 9.0 cm, height × diameter), one kernel in each pot. The plant culture mix contained Organic Culture Mix (Zhejiang Peilei Organic Fertilizer Co., Ltd., Zhengjiang, Jiangsu Province, China; N + P + K, > 2%; Organic matter content, > 35%; pH 5.5–6.5) and vermiculite at a ratio of 6:4 (w/w). The culture mix in pots (9.0 cm × 8.5 cm, diameter × height) was watered to 70–80% of the maximum water holding capacity. Finally, the pots were maintained in a growth chamber (20–25°C) under fluorescent light with the regime of 12-h light/12-h dark. When the peanut seedlings grew to reach the height of 6–8 cm, the pots with the seedlings were transferred to three plastic boxes (55 cm × 40 cm × 36.5 cm, length × width × height, ∼80 L in volume), 16 pots in each box, for the following three treatments, one box for the control treatment with the VOCs from 960 g fresh AWG medium and two other boxes for two other treatments with the VOCs from the 7-day-old AWG cultures of *S. yanglinensis* 3-10, one containing 960 g AWG culture of *S. yanglinensis* 3-10 as low dosage (12 g/L) and another one containing 2720 g AWG culture *S. yanglinensis* 3-10 as high dosage (34 g/L). The boxes were individually covered with plastic films and maintained in the growth chamber for 7 days. The seedlings were carefully up-rooted, washed under running tap water to remove soil particles. Shoot length of each seedling was measured and the seedlings were dried at 50°C for 48 h for measuring shoot the total dry weight of each seedling.

### Effect of Soil Amendment With *S. yanglinensis* 3-10 on Seedling Growth of Peanut

This is a soil amendment bioassay, aiming at determining the effect of soil amendment with *S. yanglinensis* 3-10 on growth of peanut seedlings. The 7-day-old AWG cultures of *S. yanglinensis* 3-10 (28°C) and the fresh AWG medium were air-dried at room temperature (20–25°C) and ground to fine powder, which was separately amended with the plant culture mix by a ratio of 5% (w/w). The culture mix of different treatments was loaded in pots, where pre-germinated peanut kernels were sown, one kernel in each pot and 16 pots for each treatment. The pots were maintained in the growth chamber (20–25°C, 12-h light and 12-h dark) for 30 days. Height and total dry weight of each peanut seedling were measured. This experiment was repeated two more times.

### Data Analysis

Data on colony diameter, yield of conidia produced by *A. flavus* and *A. parasiticus*, percentages of germinated conidia and length of germ tubes, disease severity, and yield of aflatoxins in peanut kernels in related experiments were separately analyzed using PROC ANOVA (analysis of variance) in the SAS software (SAS Institute, Cary, NC, United States, version 8.0, 1999). Before ANOVA, the data on conidial yield per dish was log_10_-transformed, the data on percentages of germinated conidia were transformed to numerical data by multiplication of each percentage value with 100. After ANOVA, the values were accordingly back-transformed to their original numerical forms. The means of each parameter for different treatments in each experiment were separated using least significance different (LSD) test at α = 0.05.

## Results

### The Volatile Organic Compounds Produced by *S. yanglinensis* 3-10

Gas-chromatography and mass-spectrometry analysis identified 19 major VOCs in the 3- to 14-day-old AWG cultures of *S. yanglinensis* 3-10 (Table 1 and Supplementary Figure S1). The compounds fell into seven classes, including alkenes (8), alcohols (4), aromatic naphthalenes (2), esters (2), furan (1), ketones (1), and heterocyclic benzoxepine (1). Among these VOCs, 2-MIB is the major components with the relative peak area (RPA) ranging from 12.3 to 26.6% in the 3- to 14-day-old AWG cultures, followed by 2-M2B with RPA ranging from 8.6 to 15.7%, aromatic naphthalenes with RPA ranging from 3.5 to 5.9%, 3-methyl-2-(2-methyl-2-butenyl)-furan with RPA ranging from 2.8 to 4.3%, 1-ethylideneoctahydro-7a- methyl-1H-indene with RPA ranging from 3.5 to 5.2%, *trans*-1,10-dimethyl-*trans*-9-decalinol (geosmin) with RPA ranging from 2.5 to 4.1%, and 2-PE with RPA ranging from 0.3 to 3.5%. In contrast, caryophyllene and copaene are two minor compounds with RPA lower than 1%.

**FIGURE 1:**
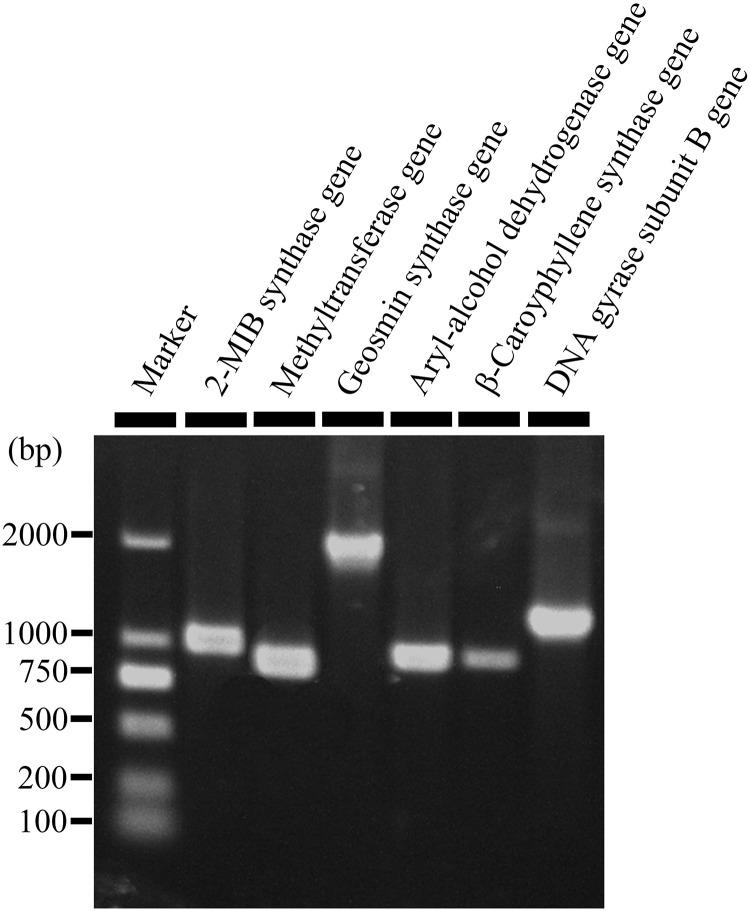
An agarose gel electrophorgram showing expression of five genes responsible for biosynthesis of 2-methyl-2-bornene (2-M2B), 2-methylisoborneol (2-MIB), 2-phenylethanol, (+)-β-caryophyllene, and geosmin in *Streptomyces yanglinensis* 3-10. Methytransferase gene and 2-MIB synthase gene are responsible for biosynthesis of 2-M2B and 2-MIB. Aryl-alcohol dehydrogenase gene, (+)-β-caryophyllene synthase gene, and geosmin synthase gene are responsible for biosynthesis of 2-phenylethanol, (+)-β-caryophyllene, and geosmin, respectively.

Production of 2-MIB, 2-M2B, β-CA, geosmin, and 2-PE by *S. yanglinensis* 3-10 was confirmed by RT-PCR detection of related genes coding for the biosynthetic enzymes (i.e., methytransferase and 2-MIB synthase for 2-MIB and 2-M2B, β-CA synthase for β-CA, aryl-alcohol dehydrogenase for 2-PE, geosmin synthase for geosmin). Results showed that the genes coding for all of these VOCs biosynthetic enzymes expressed in *S. yanglinensis* 3-10 (Figure 1).

### Antifungal Activity of the Selected VOCs

Three synthetic compounds, namely, β-CA, M2M, and 2-PE, were purchased and tested for suppression of *A. flavus* and *A. parasiticus*. The results showed that M2M and 2-PE had high antifungal activity against the two fungi. In terms of IMG, M2M showed the IC_50_ values of 7.2 and 8.0 μL/mL against *A. flavus* and *A. parasiticus*, respectively. 2-PE had even lower IC_50_ values than M2M against the two fungi, 1.2 μL/mL against *A. flavus* and 1.5 μL/mL against *A. parasiticus* (Table 2). In terms of inhibition of conidial germination, M2M showed the IC_50_ values of 0.7 and 1.2 μL/mL against *A. flavus* and *A. parasiticus*, respectively. The values were higher than those of 2-PE, which had the IC_50_ values of 51.2 μL/mL against *A. flavus* and 46.2 μL/mL against *A. parasiticus*. In contrast, β-CA had the IC_50_ values higher than 100 μL/mL in terms of IMG and conidial germination of two fungi (Table 2), suggesting that it may have no or weak antifungal activity against the two fungi.

**TABLE 2:** Values of the 50% inhibition concentration (IC_50_) of methyl 2-methylbutyrate (M2M), 2-phenylethanol (2-PE), and β-caryophyllene (β-CA) against mycelial growth and conidial germination of *Aspergillus flavus* and *A. parasiticus*.

Compound	IC_50_ (μL/mL) for		IC_50_ (μL/mL) for	
	mycelial growth^a^		conidial germination^b^	
	*A. flavus*	*A. parasiticus*	*A. flavus*	*A. parasiticus*
M2M	8.0 ± 0.5	7.2 ± 0.9	0.7 ± 0.004	1.2 ± 0.01
2-PE	1.2 ± 0.02	1.5 ± 0.2	51.2 ± 4.4	46.2 ± 15.1
β-CA	>100.0	>100.0	>100.0	>100.0

*^*a*^Mycelial growth of *A. flavus* and *A. parasiticus* was performed on potato dextrose agar in two-compartment plastic dishes at 28°C for 3 days in dark (*n* = 4). ^*b*^Conidial germination of *A. flavus* and *A. parasiticus* was performed on glucose agar in two-compartment plastic plates at 28°C for 12 h in dark (*n* = 4).*

### Antifungal Activity of the VOCs of *S. yanglinensis* 3-10 Against *Aspergillus*

Results from two bioassays in DDSs showed that the VOCs from the AWG cultures of *S. yanglinensis* 3-10 (VOCs^3–10AWG^) had strong antifungal activity against *A. flavus* and *A. parasiticus*. In the time-course bioassay, both *A. flavus* and *A. parasiticus* grew and formed significantly (*P* < 0.05) larger colonies on PDA in the control treatment with the VOCs from fresh AWG (VOCs^AWG^) than in the treatment of VOCs^3–10AWG^. At 3 dpi, *A. flavus* and *A. parasiticus* had average colony diameters of 54.2 and 46.2 mm, respectively, in VOCs^AWG^ (Table 3). The values were significantly (*P* < 0.05) lower than those in VOCs^3–10AWG^ from 3- to 14-day-old AWG cultures of *S. yanglinensis* 3-10, in which *A. flavus* had the average colony diameters smaller than 42 mm (reduced by 23–38% compared to that in VOCs^AWG^) and *A. parasiticus* had average colony diameters smaller than 29 mm (reduced by 58–70% compared to that in VOCs^AWG^). Both fungi sporulated abundantly in VOCs^AWG^ with average conidial yield reaching up to 1.3 × 10^5^ conidia/mm^2^ for *A. flavus* and to 1.0 × 10^5^ conidia/mm^2^ for *A. parasiticus*. The values were significantly (*P* < 0.05) higher than those in VOCs^3–10AWG^, in which *A. flavus* had average conidial yield lower than 0.3 × 10^5^ conidia/mm^2^ (reduced by 74–82% compared to that in VOCs^AWG^), and *A. parasiticus* had average conidial yield lower than 3.1 × 10^3^ conidia/mm^2^ (reduced by 97–98% compared to that in VOCs^AWG^).

**TABLE 3:** Antifungal activity of the volatile organic compounds (VOCs) from the autoclaved wheat grains (AWG) cultures of *Streptomyces yanglinensis* 3-10 (AWG^sy3–10^) against mycelial growth, sporulation, and conidial germination by *Aspergillus flavus* and *A. parasiticus* in double-dish sets (DDSs).

Treatment	Colony diameter	Sporulation (× 10^3^	% Germinated conidia^y^	Length of germ tubes
(source of VOCs)	(mm)^x^ (*n* = 3)	conidia/mm^2^)^x^ (*n* = 3)	(*n* = 3)	(μm)^y^ (*n* = 3)
**Against *A. flavus***				
Fresh AWG (10 g per DDS)	54.2 ± 0.9a^z^	129.2 ± 6.8a	95.4 ± 1.3a	253.5 ± 17.1a
AWG^sy3–10^ (3-day-old, 10 g per DDS)	39.2 ± 1.3bc	33.2 ± 6.5b	64.8 ± 3.2c	74.5 ± 25.6d
AWG^sy3–10^ (7-day-old, 10 g per DDS)	36.1 ± 1.0cd	23.6 ± 2.5bc	65.9 ± 3.3c	58.5 ± 16.2d
AWG^sy3–10^ (10-day-old, 10 g per DDS)	33.5 ± 3.5d	28.1 ± 7.9bc	86.5 ± 1.1b	102.8 ± 20.6c
AWG^sy3–10^ (14-day-old, 10 g per DDS)	41.8 ± 2.4b	24.6 ± 6.5bc	92.4 ± 0.8a	210.8 ± 30.7b
**Against *A. parasiticus***				
Fresh AWG (10 g per DDS)	46.2 ± 0.8a	97.3 ± 9.6a	94.6 ± 1.8a	231.8 ± 20.6a
AWG^sy3–10^ (3-day-old, 10 g per DDS)	19.5 ± 0.5b	2.6 ± 1.7b	18.3 ± 0.9c	76.8 ± 22.5b
AWG^sy3–10^ (7-day-old, 10 g per DDS)	13.7 ± 1.3c	2.2 ± 2.5b	7.3 ± 1.0d	11.4 ± 4.8c
AWG^sy3–10^ (10-day-old, 10 g per DDS)	14.3 ± 0.8c	3.1 ± 4.1b	10.6 ± 1.8d	18.5 ± 5.6c
AWG^sy3–10^ (14-day-old, 10 g per DDS)	19.2 ± 0.8b	2.2 ± 1.9b	28.9 ± 5.2b	82.8 ± 26.7b

*^*x*^Diameter of colony and sporulation were measured after incubation on PDA at 28°C for 3 days. ^*y*^Germinated conidia and length of germ tubes were measured after incubation on glucose agar at 28°C for 12 h in dark. ^*z*^Means ± SD within the same column for each fungus followed by the same letters are not significantly different (*P* > 0.05) according to least significance test.*

Results of conidial germination on GA (28°C, 12 h) showed that in VOCs^AWG^, conidia of the two fungi germinated at the rates of approximately 95%. In most treatments of VOCs^3–10AWG^, however, the conidial germination rates of both fungi were significantly (*P* < 0.05) reduced compared to that in VOCs^AWG^. *A. flavus* had average conidial germination rates ranging from 65 to 87% in VOCs^3–10AWG^ from 3-, 7-, and 10-day-old AWG cultures (reduced by 9–32% compared to that in VOCs^AWG^). However, *A. flavus* germinated by 92.4% in VOCs^3–10AWG^ from the 14-day-old AWG culture, not significantly (*P* > 0.05) different from that in VOCs^AWG^. *A. parasiticus* had the conidial germination rates ranging from 7 to 29% in VOCs^3–10AWG^ from 3-, 7-, 10-, and 14-day-old AWG cultures (reduced by 69–92% compared to that in VOCs^AWG^). Regarding germ-tube length, *A. flavus* and *A. parasiticus* had the average values of 253.5 and 231.8 μm, respectively, in VOCs^AWG^ (Table 3). The values were significantly (*P* < 0.05) reduced in the treatments of VOCs^3–10AWG^, in which *A. flavus* had average germ-tube length ranging from 58.5 to 210.8 μm (reduced by 17–76% compared to that in VOCs^AWG^), and *A. parasiticus* had average germ-tube length ranging from 11.4 to 82.8 μm (reduced by 64–95% compared to that in VOCs^AWG^).

Results from the dosage bioassay showed that the efficacy of the VOCs^3–10AWG^ from the 7-day-old AWG cultures of *S. yanglinensis* 3-10 in suppression of mycelial growth, conidial production, conidial germination, and germ-tube elongation was positively proportional to the amount of the AWG cultures of *S. yanglinensis* 3-10 applied to DDSs. For *A. flavus*, with increase in the dosage of the AWG cultures of *S. yanglinensis* 3-10 from 5 to 40 g per DDS, the suppressive efficacy was increased from 32 to 79% for colony size, from 73 to 100% for conidial yield, from 2.9 to 99% for conidial germination rates, and from 38 to 98% for germ-tube length compared to corresponding values in VOCs^AWG^. Similarly, for *A. parasiticus*, the suppressive efficacy was increased from 38 to 78% for colony size, from 95 to 100% for conidial yield, from 2.5 to 100% for conidial germination rates, and from 66 to 100% for germ-tube length compared to corresponding values in VOCs^AWG^ (Table 4).

**TABLE 4:** Antifungal activity of the volatile organic compounds (VOCs) from different dosages of the autoclaved wheat grains cultures of *Streptomyces yanglinensis* 3-10 (AWG^sy3–10^) against mycelial growth, sporulation, and conidial germination by *Aspergillus flavus* and *A. parasiticus* in double-dish sets (DDSs).

Treatment	Colony diameter	Sporulation (× 10^3^	% Germinated conidia^y^	Length of germ tubes
(source of VOCs)	(mm)^x^ (*n* = 3)	conidia/mm^2^)^x^ (*n* = 3)	(*n* = 3)	(μm)^y^ (*n* = 3)
**Against *A. flavus***				
Fresh AWG (40 g per DDS)	53.6 ± 0.3 a^z^	132.8 ± 14.6 a	95.7 ± 0.5 a	253.5 ± 13.9 a
AWG^sy3–10^ (5 g in each DDS)	36.5 ± 1.3 b	78.3 ± 28.3 b	92.9 ± 1.3 b	157.7 ± 33.5 b
AWG^sy3–10^ (10 g in each DDS)	32.0 ± 6.1 b	33 ± 16.7 c	87.7 ± 1.1 c	123.8 ± 36.2 c
AWG^sy^ ^3–10^ (20 g in each DDS)	24.6 ± 3.5 c	6.6 ± 3.1 d	16.9 ± 1.6 d	32.6 ± 10.7 d
AWG^sy3–10^ (30 g in each DDS)	18.3 ± 1.5 d	1.1 ± 1.2 d	9.3 ± 1.6 e	11.8 ± 2.5 de
AWG^sy3–10^ (40 g in each DDS)	11.2 ± 0.8 e	0.0 d	1.2 ± 0.3 f	5.8 ± 4.7 e
**Against *A. parasiticus***				
Fresh AWG (40 g per DDS)	46.3 ± 1.0 a	95.6 ± 12.4 a	94.5 ± 0.5 a	231.4 ± 18.6 a
AWG^sy3–10^ (5 g in each DDS)	28.7 ± 3.2 b	13.1 ± 8.2 bc	92.1 ± 1.9 b	77.8 ± 22.5 b
AWG^sy3–10^ (10 g in each DDS)	14.7 ± 4.5 c	25.4 ± 37.3 b	8.9 ± 0.4 c	9.9 ± 2.5 c
AWG^sy3–10^ (20 g in each DDS)	12.8 ± 0.6 c	2.3 ± 2.7 bc	3.3 ± 0.6 d	5.9 ± 4.5 c
AWG^sy3–10^ (30 g in each DDS)	11.8 ± 2.0 c	1.0 ± 2.0 bc	0.2 ± 0.02 e	3.4 ± 4.6 c
AWG^sy3–10^ (40 g in each DDS)	10.3 ± 1.0 c	0.0 c	0.0 e	0.0 c

*^*x*^The cultures of *A. flavus* or *A. parasiticus* on PDA in the presence the VOC from the fresh AWG and the 7-day-old AWG cultures of *S. yanglinensis* 3-10 (AWG^*sy*^) were incubated at 28°C for 3 days. The values are the average of three replicates. ^*y*^The conidia of *A. flavus* or *A. parasiticus* on GA in the presence the VOC from the fresh AWG and the 7-day-old AWG cultures of *S. yanglinensis* 3-10 were incubated at 28°C for 12 h. Germinated conidia and length of germ tubes were measured. The values are average of three replicates (*n* = 3). ^*z*^Means ± SD within the same column for each fungus followed by the same letter are not significantly different (*P* > 0.05) according to least significance test.*

### The Antifungal Spectrum of the VOCs From *S. yanglinensis* 3-10

Besides *A. flavus* and *A. parasiticus*, 20 other fungi and two species of *Pythium* (Oomycetes) were detected for sensitivity to VOCs^3–10AWG^ from the 7-day-old AWG culture of *S. yanglinensis* 3-10 in the DDS bioassay. Results showed that the 20 fungi and fungi-like organisms differed in response to VOCs^3–10AWG^ for mycelial growth on PDA (Table 5). Twenty-one organisms showed sensitive to VOCs^3–10AWG^, as they formed significantly (*P* < 0.05) smaller colonies on PDA in VOCs^3–10AWG^ than in VOCs^AWG^ (Supplementary Figure S16). Among these organisms, *Pyricularia oryzae* (the causal agent of rice blast) was the most sensitive fungus to VOCs^3–10AWG^, it was completely inhibited for growth in VOCs^3–10AWG^. Fifteen fungi (*Alteraria alternata*, *Bipolaris maydis*, *B. cinerea*, *Colletotrichum siamense*, *Curvularia lunata*, *Drechslera graminea*, *Fusarium moniliforme*, *Monilia fructigena*, *Mucor hiemails*, *Pestalotia theae*, *R. solani*, *Rhizopus stolonifer*, *Sclerotinia minor*, *S. sclerotiorum*, and *Sclerotium rolfsii*) and *Pythium apanidermatum* showed moderately sensitive to VOCs^3–10AWG^. Size of the colonies formed by these organisms in the presence of VOCs^3–10AWG^ was reduced by 39–85% compared to that in VOCs^AWG^. Two fungi (*Gaeumanomyces graminis* var. *tritici* and *Fusarium oxysporum* f. sp. *vasinfectum*) were slightly sensitive to VOCs^3–10AWG^, size of the colonies formed in VOCs^3–10AWG^ was reduced by 14.2 and 26.0%, respectively, compared to that in VOCs^AWG^. In contrast, *Pythium ultimum* was insensitive to VOCs^3–10AWG^, colony size of *P. ultimum* was reduced by 1.4% in VOCs^3–10AWG^, compared to that in VOCs^AWG^.

**TABLE 5:** The antifungal spectrum of the volatile organic compounds (VOCs) emitted from the cultures of *Streptomyces yanglinensis* 3-10 on autoclaved wheat grains.

Fungus	Colony diameter (mm) (Mean ± SD)		Inhibition of growth (%)	Culture conditions (temperature, time^1^)
	−VOCs	+ VOCs		
**Oomycota**				
*Pythium apanidermatum*	56.2 ± 1.3	34.2 ± 0.8	39.2 ± 1.4	20°C at 4 dpi
*Pythium ultimum*	82.5 ± 0.5	81.0 ± 1.0	1.4 ± 0.7	20°C at 1 dpi
**Zygomycota**				
*Mucor hiemails*	64.2 ± 0.6	30.8 ± 6.5	51.9 ± 10.2	20°C at 3 dpi
*Rhizopus stolonifer*	62.8 ± 1.5	26.3 ± 6.8	58.1 ± 10.9	20°C at 1 dpi
**Ascomycota**				
*Alteraria alternata*	51.2 ± 4.4	10.8 ± 5.7	78.9 ± 1.1	25°C at 3 dpi
*Botrytis cinerea*	62.6 ± 1.6	12.5 ± 0.5	80.1 ± 0.8	20°C at 2 dpi
*Bipolaris maydis*	23.2 ± 3.1	6.5 ± 0.5	71.9 ± 2.2	28°C at 5 dpi
*Colletotrichum siamense*	46.8 ± 0.8	22.8 ± 0.8	51.6 ± 1.1	28°C at 3 dpi
*Curvularia lunata*	55.0 ± 5.0	33.0 ± 4.4	39.9 ± 7.9	25°C at 3 dpi
*Drechslera graminea*	67.7 ± 2.5	29.5 ± 6.9	56.4 ± 10.3	20°C at 3 dpi
*Fusarium moniliforme*	80.3 ± 1.4	37.0 ± 9.6	54.2 ± 11.9	20°C at 3 dpi
*F. oxysporum* f.sp. *vasinfectum*	60.2 ± 0.3	44.5 ± 0.5	26.0 ± 0.8	20°C at 3 dpi
*Gaeumanomyces graminis* var. *tritici*	71.7 ± 1.4	61.3 ± 1.3	14.2 ± 1.4	25°C at 2 dpi
*Monilia fructigena*	70.0 ± 1.0	11.0 ± 2.2	84.3 ± 3.1	20°C at 3 dpi
*Pestalotia theae*	72.0 ± 1.7	24.0 ± 8.0	66.7 ± 1.2	20°C at 3 dpi
*Pyricularia oryzae*	35.7 ± 1.2	6.0	100	20°C at 5 dpi
*Sclerotinia minor*	67.2 ± 0.8	16.0 ± 0.9	76.2 ± 1.3	20°C at 2 dpi
*Sclerotinia sclerotiorum*	76.3 ± 1.9	12.7 ± 1.0	83.4 ± 1.4	20°C at 2 dpi
**Basidiomycota**				
*Rhizoctonia solani*	76.5 ± 0.5	22.8 ± 0.8	70.4 ± 0.7	20°C at 2 dpi
*Sclerotium rolfsii*	75.2 ± 0.8	15.5 ± 1.0	79.3 ± 1.4	20°C at 3 dpi

*^1^dpi = days post-incubation.*

### *Streptomyces* VOCs-Mediated Suppression of *Aspergillus* Colonization of Peanut Kernels and Production of Aflatoxins

**FIGURE 2:**
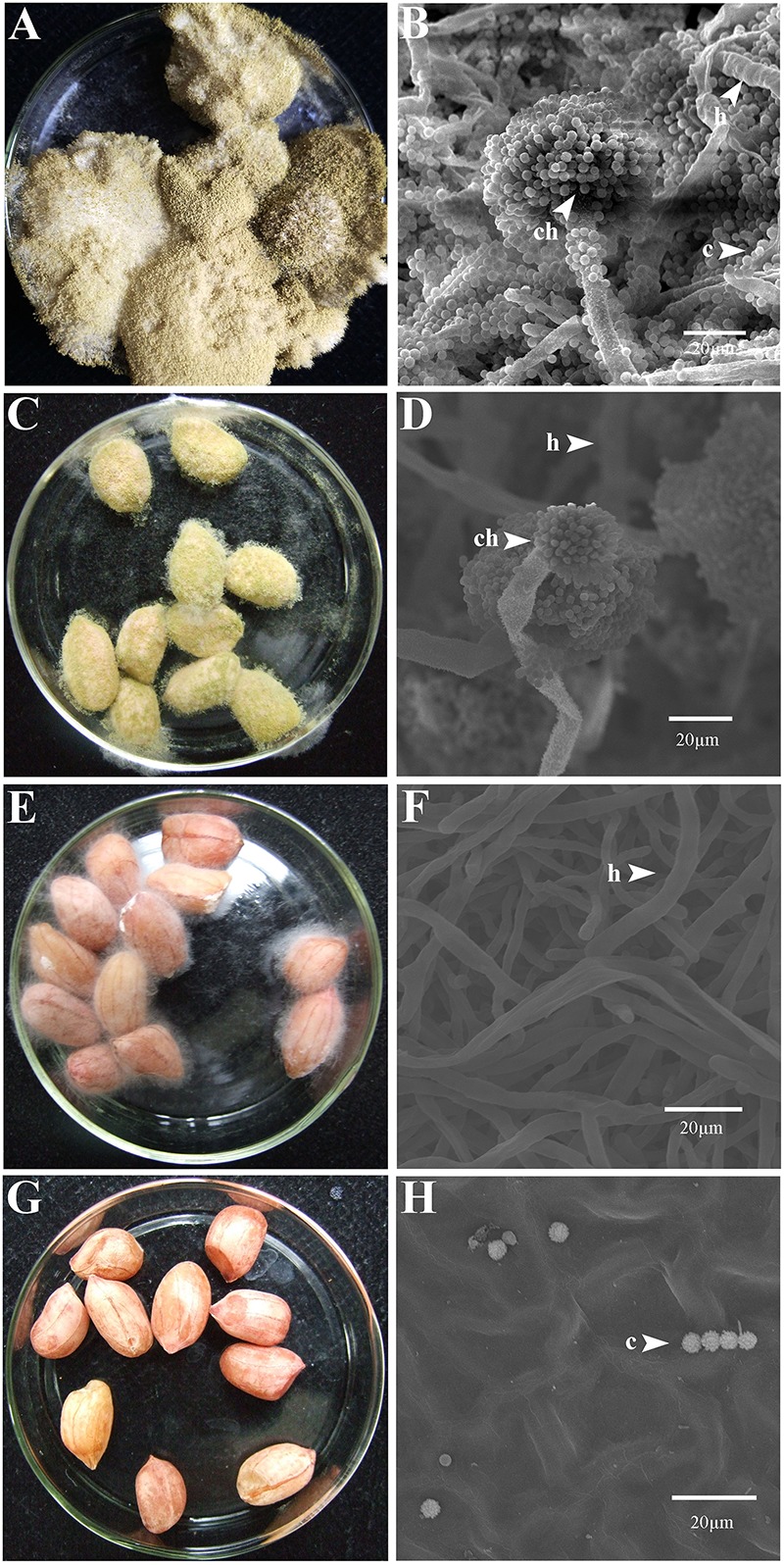
Colonization of peanut kernels by *Aspergillus flavus* in the presence of the volatiles from the fresh AWG medium (control) and the AWG cultures of *Streptomyces yanglinensis* 3-10 at different dosages. **(A,B)** Control, peanut kernels colonized by *A. flavus*. Note the dense aerial hyphae and vigorous sporulation on the kernel surface with a yellow color. A scanning electron microscopic (SEM) image showed hyphae (h), a conidiophore head (ch), and abundant conidia (c). **(C,D)** AWG culture of *S*. *yanglinensis* 3-10 at 17 g/L, a dish with peanut kernels colonized by *A. flavus*. Note relatively sparse aerial hyphae and sporulation on the kernel surface with a yellow color. The SEM image showed hyphae (h), conidiophores head (ch), and conidia (c). **(E,F)** AWG culture of *S*. *yanglinensis* 3-10 at 52 g/L, a dish with peanut kernels colonized by *A. flavus*. Note sparse aerial hyphae of a white color without visible sporulation on the kernel surface. The SEM image showed abundant hyphae. **(G,H)** AWG culture of *S. yanglinensis* 3-10 at 86 g/L, a dish with peanut kernels without visible colonization by *A. flavus* on the kernel surface. The SEM image showed that the inoculated conidia of *A. flavus* failed to germinate.

**FIGURE 3:**
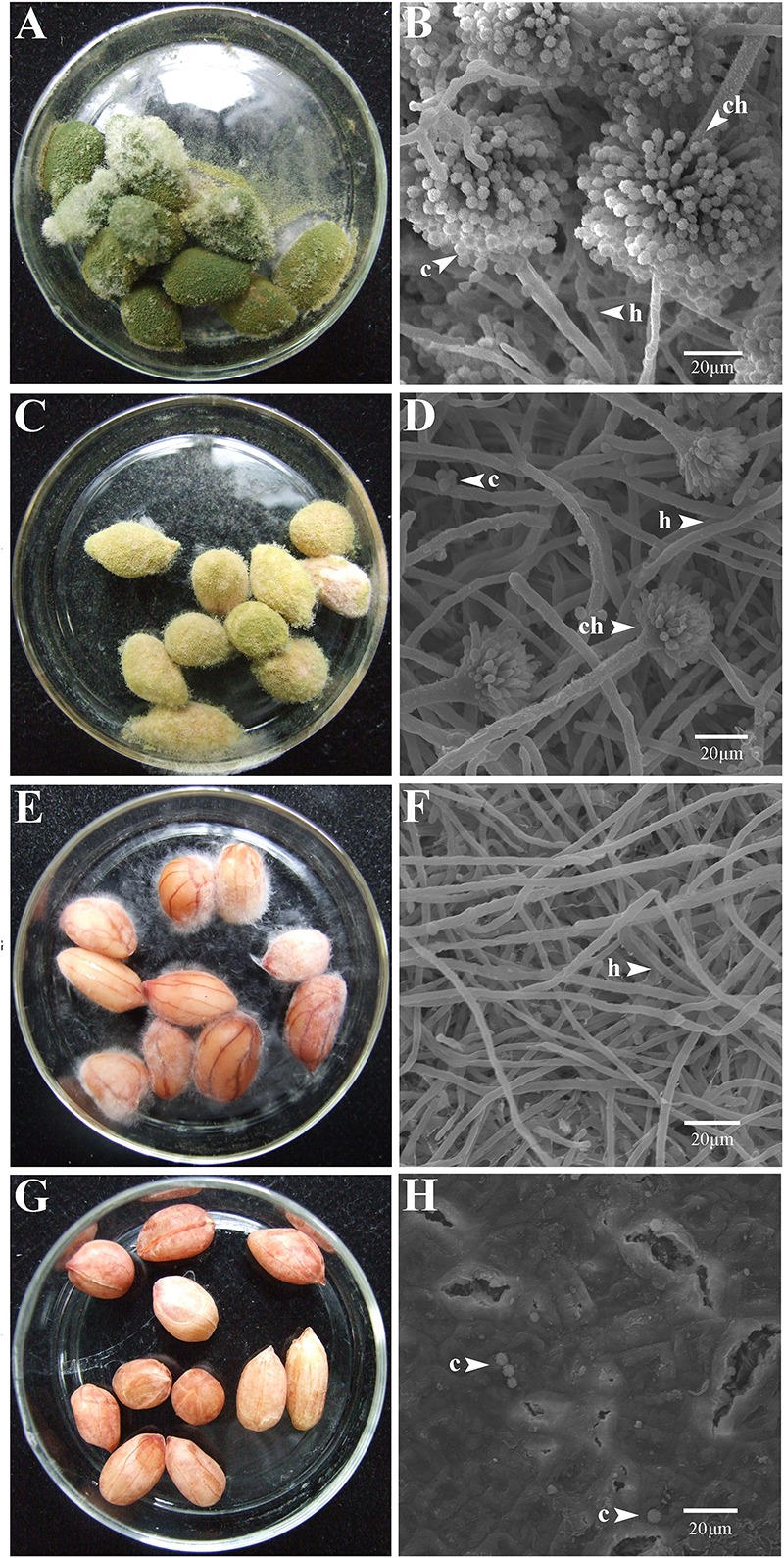
Colonization of peanut kernels by *Aspergillus parasiticus* in the presence of the volatiles from the fresh AWG medium (control) and the AWG cultures of *Streptomyces yanglinensis* 3-10 at different dosages. **(A,B)** Control, peanut kernels colonized by *A. flavus*. Note the dense aerial hyphae and vigorous sporulation on the kernel surface with a green color. A scanning electron microscopic (SEM) image showed hyphae (h), a conidiophore head (ch), and abundant conidia (c). **(C,D)** AWG culture of *S*. *yanglinensis* 3-10 at 17 g/L, a dish with peanut kernels colonized by *A. parasiticus*. Note relatively sparse aerial hyphae and sporulation on the kernel surface with a yellowish green color. The SEM image showed hyphae (h), conidiophores head (ch), and conidia (c). **(E,F)** AWG culture of *S*. *yanglinensis* 3-10 at 52 g/L, a dish with peanut kernels colonized by *A. parasiticus*. Note sparse aerial hyphae of a white color without visible sporulation on the kernel surface. The SEM image showed abundant hyphae. **(G,H)** AWG culture of *S. yanglinensis* 3-10 at 86 g/L, a dish with peanut kernels without visible colonization by *A. parasiticus* on the kernel surface. The SEM image showed that the inoculated conidia of *A. flavus* failed to germinate.

**FIGURE 4:**
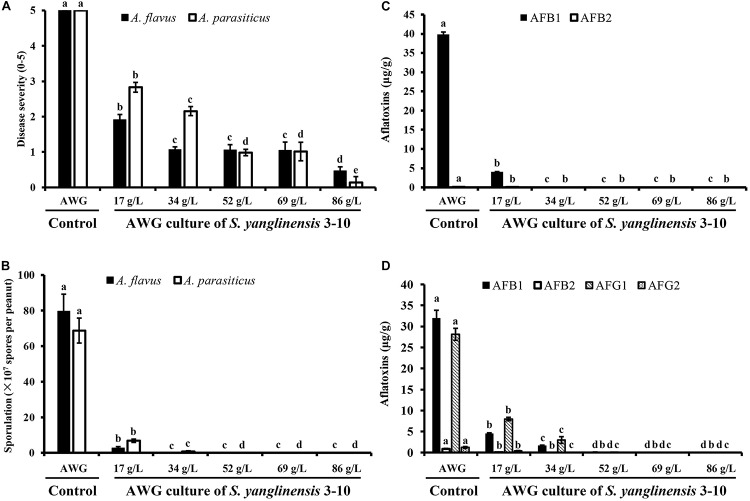
Four histograms showing on disease severity, conidial production and aflatoxin production by *Aspergillus flavus* and *A. parasiticus* in peanut kernels (28°C, 7 dpi). **(A)** Disease severity on peanut kernels infected with *A. flavus* and *A. parasiticus*; **(B)** Conidial sporulation by *A. flavus* and *A. parasiticus* on peanut kernels. **(C)** Yield of aflatoxin B produced by *A. flavus* on peanut kernels. **(D)** Yield of aflatoxins (AFB_1_, AFB_2_, AFG_1_, and AFG_2_) produced by *A. parasiticus* on peanut kernels. In each histogram, means ± S.D. labeled with the same letters indicate no significant difference (*P* > 0.05) according to Least Significant Difference (LSD) test.

At 7 dpi (28°C), the *Aspergillus*-inoculated peanut kernels in the treatment of VOCs^AWG^ were fully colonized by *A. flavus* and *A. parasiticus* on the kernel surface with the disease severity reaching up to 5 (Figures 2–4). Both fungi formed dense and compact mycelial masses, and sporulated abundantly with average conidial yield reaching up to 10^8^ conidia per kernel. In contrast, in treatments of VOCs^3–10AWG^ at 17, 34, 52, 69, and 86 g/L (designated as VOCs^3–10AWG^-17, VOCs^3–10AWG^-34, VOCs^3–10AWG^-52, and VOCs^3–10AWG^-69, and VOCs^3–10AWG^-86, respectively), colonization and sporulation on the kernels by the two fungi were suppressed. The average disease severity values were reduced to 0.5–1.9 and 0.1–2.8 on the *A. flavus*- and *A. parasiticus*-inoculated kernels, respectively. The average conidial yield on *A. flavus-*inoculated kernels was reduced to 2.7 × 10^7^ conidia per kernel in VOCs^3–10AWG^-17, and to 0 in VOCs^3–10AWG^-34, VOCs^3–10AWG^-52, VOCs^3–10AWG^-69, and VOCs^3–10AWG^-86. The average conidial yield on *A. parasiticus-*inoculated kernels was reduced to 6.8 × 10^7^ and 7.3 × 10^6^ conidia per kernel in VOCs^3–10AWG^-17 and VOCs^3–10AWG^-34, respectively, and to 0 in VOCs^3–10AWG^-52, VOCs^3–10AWG^-69, and VOCs^3–10AWG^-86.

Production of aflatoxins (AFB_1_, AFB_2_, AFG_1_, AFG_2_) in *Aspergillus*-inoculated peanut kernels were quantified using the LC-MS method. Two aflatoxins (AFB_1_, AFB_2_) were detected in *A. flavus*-inoculated kernels. In the control treatment with VOCs^AWG^, the average yield of AFB_1_ and AFB_2_ reached up to 39.9 and 0.1 μg/g kernel, respectively (Figure 4). However, in the treatments of the VOCs^3–10AWG^-17, VOCs^3–10AWG^-34, VOCs^3–10AWG^-52, and VOCs^3–10AWG^-69, and VOCs^3–10AWG^-86, production of AFB_1_ and AFB_2_ was significantly (*P* < 0.05) suppressed compared to that in VOCs^AWG^. In VOCs^3–10AWG^-17, the average yield of AFB_1_ and AFB_2_ was 4.1 and 0.002 μg/g kernel, respectively (reduced by 89.7 and 98%, respectively, compared to that in VOCs^AWG^). In VOCs^3–10AWG^-34, VOCs^3–10AWG^-52, VOCs^3–10AWG^-69, and VOCs^3–10AWG^-86, production of AFB_1_ and AFB_2_ was not detected at all. Production of four aflatoxins, namely, AFB_1_, AFB_2_, AFG_1_, and AFG_2_, was detected in *A. parasiticus*-inoculated peanut kernels. In VOCs^AWG^, the average yield of AFB_1_, AFB_2_, AFG_1_, and AFG_2_ reached up to 31.9, 0.9, 28.1, and 1.2 μg/g kernel, respectively. In VOCs^3–10AWG^-17, the average yield of AFB_1_, AFB_2_, AFG_1_, and AFG_2_ was suppressed to 4.4, 0.01, 7.9, and 0.4 μg/g kernel, respectively (reduced by 67–99% compared to that in VOCs^AWG^). In VOCs^3–10AWG^-34, the average yield of the four alfatoxins was suppressed to 1.5, 0.0, 2.9, and 0.0 μg/g kernel, respectively (reduced by 90–100% compared to that in VOCs^AWG^). In VOCs^3–10AWG^-34, VOCs^3–10AWG^-52, VOCs^3–10AWG^-69, and VOCs^3–10AWG^-86, production of the four aflatoxins was not detected at all.

### *Streptomyces* VOCs-Mediated Suppression of Expression of Aflatoxins Biosynthesis Genes in *Aspergillus*

**FIGURE 5:**
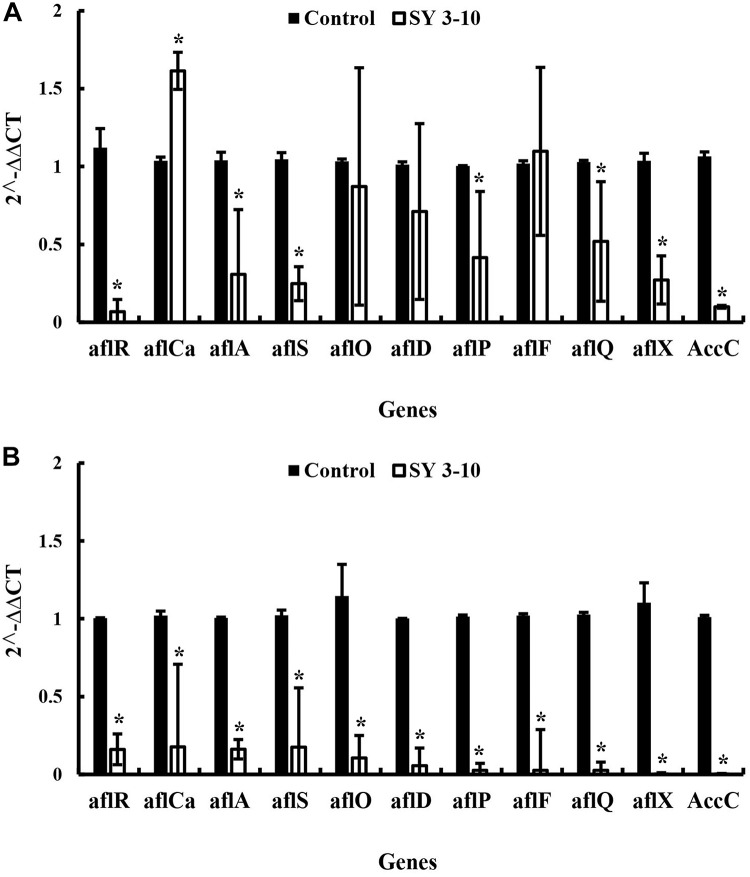
Expression of the genes for biosynthesis of the aflatoxins in *A. flavus* **(A)** and *A. parasiticus* **(B)** in the presence and absence of the VOCs of *S. yanglinensis* 3-10 (SY3-10). *significant difference at *p* < 0.05 in comparison to the control treatment according the Student’s *t*-test.

The results showed that the VOCs from *S. yanglinensis* 3-10 reduced expression of the aflatoxins biosynthesis genes. In *A. flavus*, expression of seven (*aflR*, *aflA*, *aflS*, *aflP*, *aflQ*, *aflX*, and *AccC*) out of the 11 tested genes was reduced by 1.18–20.17 folds in the treatment of the VOCs, compared to the expression level of each gene in the control treatment (Figure 5A). In *A. parasiticus*, expression of all of the 11 genes reduced with the treatment of VOCs by 5.7–537.2 folds, compared to the expression level of each gene in the control treatment (Figure 5B).

### Effects of the *Streptomyces* VOCs and Soil Amendment With *S. yanglinensis* 3-10 on Growth of Peanut Seedlings

In the VOCs-fumigation bioassay, the peanut seedlings were exposed for 7 days (20–25°C) to the VOCs either from the fresh AWG (VOCs^AWG^ as control, 12 g/L) or from the AWG cultures of *S. yanglinensis* 3-10 (VOCs^3–10AWG^, 12 or 34 g/L). The results showed that the peanut seedlings in both treatments grow normally. The two treatments did not significantly differ (*P* > 0.05) in average seedling height and total seedling dry weight per seedling (Supplementary Table S4 and Supplementary Figure S17). In the soil amendment bioassay, the cultural mix was amended either with the powder of the un-colonized AWG medium (control) or the powder of *S. yanglinensis*-colonized AWG (5%, w/w). The 30-day-old seedlings (25°C) in both treatments did not differ significantly (*P* > 0.05) from each other in average seedling height and total dry weight per seedling (Supplementary Table S4). These results suggest that the VOCs from *S. yanglinensis* 3-10 and soil amendment with *S. yanglinensis* 3-10 may have no harmful effect on peanut seedling growth.

## Discussion

Control of *A. flavus* and *A. parasiticus* in food/feed production is fundamentally important due to their aflatoxins producing ability. Effective control of food/feed contamination by *Aspergillus* spp. can be achieved by minimizing the amount of the primary inoculum in the field and/or under the storage conditions (Abdel-Kareem et al., 2019). Except for the traditional control methods, the microbial VOCs used as fumigant may be a promising alternative method. *Streptomyces* spp. are well known as producers of hydrolytic enzymes and antifungal metabolites with inhibitory effects against many plant pathogenic fungi including *Aspergillus* species (Mander et al., 2016; Shakeel et al., 2018). Use of the VOCs from *Streptomyces* for control of *Aspergillus* contamination in peanut kernels has not been reported so far. In this study, we demonstrated that VOCs from *S. yanglinensis* 3-10 could prevent *Aspergillus* contamination on peanut kernels under the storage conditions. The VOCs also showed antifungal activity against mycelial growth of 20 fungal species and one Oomycete (Table 5). In these fungi, except *Aspergillus*, the fungi in the genera of *Mucor*, *Rhizopus*, *Botrytis*, *Monilia*, and *Pythium* cause rot disease on fruits and vegetables during storage. These results suggest that the VOCs from *S. yanglinensis* 3-10 have a promising potential used as a biofumigant with a broad antifungal spectrum, and can be potentially used in food/feed postharvest disease control.

The VOCs from *S. yanglinensis* 3-10 exhibited suppressive effect on *A. flavus* and *A. parasiticus in vitro* assay and on peanut kernels under storage condition. In the *in vitro* assay, 7- and 10-day-old AWG culture of *S. yanglinensis* 3-10 showed the great inhibitory activity to mycelial growth, sporulation, conidia germination, and germ-tube elongation of *A. flavus* and *A. parasiticus* (Table 3). In the *in vivo* assay, SEM observation showed that the conidia of *A. flavus* and *A. parasiticus*-inoculated on the surface of peanut kernels hardly germinated in the treatment with high dosages of the AWG cultures of *S. yanglinensis* 3-10 (Figures 2, 3), the average disease severity was significantly decreased under the fumigation by the VOCs from *S. yanglinensis* 3-10 (Figure 4A). In the presence of high dosages of the AWG cultures of *S. yanglinensis* 3-10 (52–86 g/L), both *A. flavus* and *A. parasiticus* hardly grew, sporulates and produces aflatoxin (Figures 4B–D). It is well known that sporulation cultures of *A. flavus* and *A. parasiticus* are capable of producing aflatoxins (Seenappa and Kempton, 1980) and the conidia from *A. flavus* and *A. parasiticus* could be the major source of the primary inoculum (Diener et al., 1987). The VOCs from *S. yanglinensis* 3-10 exhibited great ability to prevent formation of the primary inoculum by inhibition of sporulation by *A. flavus* and *A. parasiticus*. Microbial VOCs can easily diffuse in an airtight condition (Wang et al., 2013), and the antifungal VOCs can inhibit the growth of plant pathogenic fungi by avoiding direct contact with the pathogens and hosts. Therefore, using VOCs from microbes for controlling food/feed postharvest diseases could be viewed as a safely and environment-friendly measure. This suggests that the VOCs from *S. yanglinensis* 3-10 could be used as a biofumigant agent for storage of peanut kernels.

Nineteen major VOCs from *S. yanglinensis* 3-10 were detected and identified by SPME-GC-MS analysis (Table 1), most of these compounds were detected in actinomycetes (Schöller et al., 2002; Wilkins and Schöller, 2009). The component 2-MIB was the main volatile among the VOCs emitted by *S. yanglinensis* 3-10 and trans-1,10-Dimethyl-trans-9-decalinol (geosmin) was also detected. Both 2-MIB and geosmin are tertiary alcohols with earthy smelling and the principal odor components of soil (Buttery and Garibaldi, 1976). These two VOCs can be found in *Streptomyces*, cyanobacteria, and fungi such as *Penicillium* and *Aspergillus* species (Jüttner and Watson, 2007). The VOC M2M has been detected in apple cortex (Leisso et al., 2015), strawberry (Song et al., 2017), chamomile oil (Bail et al., 2009), co-culture of *Enterobacter cloacae* and *Pseudomonas aeruginosa* (Lawal et al., 2018), and also in the cultures of actinomycetes (Dickschat et al., 2011). M2M has been reported to have a moderate inhibitory effect against *Staphylococcus aureus*, *Enterococcus faecali*, *P. aeruginosa*, *Proteus vulgaris*, *Klebsiella pneumonia*, *Salmonella* sp., and *Candida albicans* (Bail et al., 2009). In our study, M2M showed high inhibitory activity against germination of conidia of *A. flavus* and *A. parasiticus* (Table 2). M2M and its homologs including 2-methylbutyl acetate, 2-methylbutyl 2-methylbutyrate, 2-methylbutyl angelate, and ethyl 2-methylbutyrate have been demonstrated to have inhibitory activity against fungi and bacteria, but the antimicrobial mechanism of these compounds has not been studied (Bail et al., 2009; Guo et al., 2019).

The 2-PE is one of the most widespread aromatic VOCs (Schulz and Dickschat, 2007). In the VOCs from yeast (Huang et al., 2011), plant endophytic fungi *M. albus* (Strobel et al., 2001), *M. crispans* (Mitchell et al., 2010), and *Streptomyces* (Li et al., 2010), production of 2-PE was detected and the compound showed antifungal activity against many plant pathogenic fungi. Previous studies reported that 2-PE from *Pichia anomala* inhibit mycelial growth and expression of aflatoxin biosynthetic genes in *A. flavus* (Hua et al., 2014; Chang et al., 2015). It showed a lethal effect against bacteria and fungi at very low concentrations (0.3–0.5%) (Chang et al., 2015). At the sublethal concentration, 2-PE was found to reduce rates of mycelial growth and conidial gerimination (Chang et al., 2015). In previous studies, 2-PE was exhibited inhibitory effects on biosynthesis of DNA, amino acid and protein biosynthesis, and disruption of subcellular changes in membrane integrity (Chang et al., 2015). In our study, we found that 2-PE was more effective on suppression of mycelial growth than on conidial germination (Table 2).

This study found that the VOC β-CA showed weakly antifungal activity against mycelial growth and conidial germination of *A. flavus* and *A. parasiticus* (Table 2). Previous studies demonstrated β-CA can promote plant growth (Minerdi et al., 2011). Yamagiwa et al. (2011) reported that β-CA could also significantly enhance growth of *Brassica campestris* and resistance to anthracnose disease caused by Colletotrichum *higginsianum*. It seems that the VOC from *S. yanglinensis* 3-10 may have potential to enhance plant growth and synergistically to exhibit antifungal activity. In the VOCs from *S. yanglinensis* 3-10, 3-methyl-2-(2-methyl-2-butenyl)-furan (rosefuran) was detected in 7, 10, and 14-day-old AWG cultures, but not in 3-day-old AWG culture, the content was increased with the elongation of the culture time. Rosefuran was a minor, but an important olfactive ingredient of Bulgaria rose, *Elsholtzia ciliata* oil (Tsukasa, 1989), and essential oil of *Perilla ocimoides* (Misra and Husain, 1987). Mori et al. (1998) reported that rosefuran was a sex pheromone of an acarid mite. To our knowledge, this is the first discovery of rosefuran in actinomycetes.

Sulfur-containing VOCs dimethyl disulfide (DMDS) and dimethyl trisulfide (DMTS) were not detected in the VOCs from *S. yanglinensis* 3-10. They were reported as the main VOC component with a broad spectrum of antifungal activity in microbes such as *Alcaligenes*, *Pseudomonas*, *Shewanella*, and *Streptomyces* (Li et al., 2010; Gong et al., 2015, 2019). DMDS and DMTS have a repelling activity, which may limit its use as biofumigant. Until now, a few of VOCs are reported inhibitory activity against *A. flavus* and *A. parasiticus* (Gong et al., 2019). In our study, a novel VOC M2M showed inhibition of conidial germination, and 2-PE showed suppressive effect on mycelial growth *in vitro*, but the relative content of M2M and 2-PE was below 5%. These results indicated that inhibitory effect of the VOCs from *S. yanglinensis* 3-10 against *Aspergillus* may have synergistic effect among these volatiles. In *Streptomyces*, the biosynthesis pathway for 2-MIB, 2-M2B, geosmin, and β-CA have been studied (Supplementary Figure S2). Komatsu et al. (2008) identified 2-MIB synthase encoded by SCO7700 gene in *Streptomyces coelicolor* A3(2), SGR1269 gene in *Streptomyces griseus* IFO13350, SCAB 5041 gene in *Streptomyces scabiei* 87.22 (directly catalyzed the biosynthesis of 2-MIB and 2-M2B). Jiang et al. (2007) reported geosmin synthase encoded by SCO6073 gene in *S. coelicolor* A3(2) generated geosmin from farnesyl diphosphate. Nakano et al. (2011) characterized a terpenoid cyclase encoded by gene SGR2079 in *S. griseus* IFO13350 was responsible for biosynthesis of β-CA and caryolan-1-ol. In *Enterobacter* sp. CGMCC 5087, 2-PE was generated by aryl-alcohol dehydrogenase through the phenylpyruvate pathway. The amino acid alignment showed the conserved motifs in these key genes in our study are identical to the amino acid sequences in reference genes (Supplementary Figures S3–S12). It is supposed that the function of these key genes were similar to the reported genes.

The VOCs from *S. yanglinensis* 3-10 could suppress expression of the aflatoxin biosynthesis genes. The *aflR* gene in *A. flavus* and *A. parasiticus* was downregulated by 20.17 and 6.26 folds, respectively, compared to the expression level of this gene in the control treatment. The *aflS* gene in *A. flavus* and *A. parasiticus* was also downregulated by 4.33 and 6.12 folds, respectively, compared to the expression level of this gene in the control treatment. The *aflR* gene is required for transcriptional activation of aflatoxin biosynthesis and the *aflS* gene is a transcriptional enhancer. Previous studies demonstrated that *aflR* gene and *aflS* gene might be involved in regulation of other genes in the aflatoxin biosynthesis pathway; thus, it can directly manipulate aflatoxins biosynthesis (Flaherty and Payne, 1997; Cary et al., 2000; Amare and Keller, 2014). In our study, the expression of these two genes was significantly reduced and the aflatoxin quantification result was further proved the importance of *aflR* and *aflS* expression in aflatoxin biosynthesis. After treatment with the VOCs from *S. yanglinensis* 3-10, the biomass and expression of the aflatoxins biosynthesis genes in *A. flavus* and *A. parasiticus* were decreased, these findings may explain reduction of aflatoxins production by the VOCs produced by *S. yanglinensis* 3-10.

*Aspergillus* is commonly found in soil and crop debris. In biological control of *A. flavus* and *A. parasiticus*, using atoxigenic *Aspergillus* isolates can reduce aflatoxin contamination (Abbas et al., 2011; Amaike and Keller, 2011). In the field, soil amendment with the conidia of atoxigenic *A. flavus* and *A. parasiticus* or use in irrigation could reduce aflatoxin concentrations in peanuts (Dorner et al., 1992; Dorner and Horn, 2007), cotton (Cotty, 1994), and maize (Dorner, 2009). Our results indicate that the VOCs emitted by *S. yanglinensis* 3-10 and soil amendment with the AWG cultures of *S. yanglinensis* 3-10 did not show harmful effects on peanut seedling growth. *Streptomyces* spp. are soil dwelling bacteria, they can grow and produce versatile secondary metabolites in soil. *S. yanglinensis* 3-10 was proved to produce antifungal metabolites which showed inhibitory effects on *A. flavus* growth and aflatoxin production and other plant pathogenic fungi (Lyu et al., 2017; Shakeel et al., 2018). It is an acidophilic species with high adaptation ability in acidic soils in southern China. Considering the ability to produce antifungal metabolites and the VOCs, *S. yanglinensis* 3-10 can be developed as a biocontrol agent applied in the field for control of *Aspergillus* as well as other plant pathogenic fungi and Oomycetes or for prevention of food/feed contamination under the storage conditions.

## Conclusion

The VOCs produced by *S. yanglinensis* 3-10 displayed a wide antifungal spectrum, including postharvest and soilborne plant pathogenic fungi, such as *A. flavus* and *A. parasiticus*. The VOCs also showed an inhibitory effect on production of the aflatoxins by *A. flavus* and *A. parasiticus* in peanut kernels through suppression of colonization by the two fungi and down-regulation of expression of the aflatoxin biosynthesis genes in the two fungi. This study further demonstrated that *S. yanglinensis* 3-10 is a promising potential with versatile mechanisms in suppression of plant pathogenic fungi, including *A. flavus* and *A. parasiticus*.

## Data Availability Statement

Publicly available datasets were analyzed in this study. This data can be found here: GenBank accession numbers for nucleotide sequences: MK861971, MK861972, MK861973, MK861974, MK861975, and MK861976.

## Author Contributions

AL and LY designed research. AL performed research and analyzed the data of SPME-GC-MS. JZ and MW provided new agents and analyzed the data. AL and GL wrote the manuscript.

## Conflict of Interest

The authors declare that the research was conducted in the absence of any commercial or financial relationships that could be construed as a potential conflict of interest.

## Footnotes

1 https://antismash.secondarymetabolites.org

2 https://www.kegg.jp
