# Supplementary material for: High Efficacy of the Volatile Organic Compounds of Streptomyces yanglinensis 3-10 in Suppression of Aspergillus Contamination on Peanut Kernels
Source: Front Microbiol. 2020 Feb 6;11:142. doi: 10.3389/fmicb.2020.00142 (PMC7015977; doi:10.3389/fmicb.2020.00142)
Supplement: Supplementary file 1 [file Data_Sheet_1.pdf]

High efficacy of the Volatile Organic Compounds of *Streptomyces yanglinensis* 3-10 in Suppression of *Aspergillus* Contamination on Peanut Kernels

Ang Lyu[1,2,3], Long Yang[1,2], Jing Zhang[1,2], Mingde Wu[1,2], and Guoqing Li[1,2]*

[1]State Key Laboratory of Agricultural Microbiology and Key Laboratory of Plant Pathology of Hubei Province, [2]Huazhong Agricultural University, Wuhan 430070, China

[3]School of life science and technology, Hubei Engineering University, Xiaogan 432000, China

*****Correspondence: Dr. Guoqing Li, E-mail: guoqingli@mail.hzau.edu.cn

I. Supplementary Tables

Supplementary Table S1. List of the microbial isolates used in this study.

Test fungus and isolates	Host and/or origin[*]
Actinomycetes	
Streptomyces yanglinensis 3-10	Rice (*Oryza sativa*), HZAU
Fungi	
Alteraria alternata TBS-3	Tobacco (*Nicotiana tabacum*), HZAU
Aspergillus flavus NRRL3357	Peanut (*Arachis hypogaea*), NRRL
Aspergillus parasiticus MO527	Rice Rice (*Oryza sativa*), CGMCC
Botrytis cinerea RoseBC-3	Rose (*Rosa chinensis*), HZAU
Bipolaris maydis MB-1	Maize (*Zea mays*), HZAU
Curvularia lunata CFW-4	Rice (*Oryza sativa*), HZAU
Colletotrichum siamense Nj-2	Strawberry (*Fragaria×ananassa*), HAAS
Drechslera graminea BGW014	Barley (*Hordeum vulgare*), HZAU
Fusarium moniliforme WF-3	Rice (*Oryza sativa*) , HZAU
Fusarium oxysporum f. sp. *vasinfectum* FO-25	Cotton (*Gossypium hirsutum*), HAAS
Gaeumanomyces graminis var. *tritici*	Wheat (*Triticum aestivum* L.), HnAAS
Monilia fructigena BT15-56	Peach (*Amygdalus persica*), HZAU
Mucor hiemails WHT-1	Tomato (*Lycopersicon esculentum*), HZAU
Pestalotia theae DMZ21	Rice (*Oryza sativa*), HZAU
Pyricularia oryzae PB-1	Rice (*Oryza sativa*), HZAU
Rhizoctonia solani WH-1	Rice (*Oryza sativa*), HZAU
Rhizopus stolonifer WHS-1	Strawberry (*Fragaria × ananassa*), HZAU
Sclerotinia minor Lc-45	Lettuce *(Lactuca sativa)*, HZAU
Sclerotinia sclerotiorum Ss-1	Oilseed rape (*Brassica napus*), HZAU
Sclerotium rolfsii B-1	Plumepoppy (*Macleaya cordata*), HZAU
Oomycetes	
Pythium ultimum WLOW2F	Watermelon (*Citrullus lanatus*), WAAST
Pythium aphanidermatum Py-xg	Watermelon (*Citrullus lanatus*), WAAST

[*]NRRL = Agricultural Research Service Culture Collection, United States Department of Agriculture; CGMCC = China General Microbiological Culture Collection Center, Beijing, China: HbAAS = Hubei Academy of Agricultural Sciences, Wuhan, China; HnAAS = Henan Academy of Agricultural Sciences, Zhengzhou, China; HZAU = Huazhong Agricultural University, Wuhan, China: WAAST = Wuhan Academy of Agricultural Science and Technology, Wuhan, China.

Supplementary Table S2. List of the oligonucleotide primers for RT- PCR amplification in this study.

Gene	GenBank Acc. No.	Sequence (5’-3’)	Thermal program in RT-PCR	Size of the RT-PCR product
2-MIB synthase gene	MK861971	Forward: TGGTTGTGGATC TCGACACTG Reverse: ACCTCGTCACGG CCTACTAC	Initial denaturation: 95℃ for 5 min; 32 cycles: 95℃ for 30s, 56℃ for 30s, 72℃ for 1 min 15s;	1107 bp
Methyltransfer-ase gene	MK861972	Forward: AGAAACGGTTCT TCGACCCC Reverse: CTGAGGAGAACC CGGTCAAC	Initial denaturation: 95℃ for 5 min; 32 cycles: 95℃ for 30s, 56℃ for 30s, 72℃ for 1 min;	876 bp
Geosmin synthase gene	MK861973	Forward: CTCGTGCCAGGT GAGGATGCC Reverse: CGCGTTCTACGT GCCGTACC	Initial denaturation: 95℃ for 5 min; 32 cycles: 95℃ for 30s, 56℃ for 30s, 72℃ for 2 min 15s;	2277 bp
Aryl-alcohol dehydrogenase gene	MK861974	Forward: GATGTGCCGGAC CGACC Reverse: GATGAGCTGGTC GAAGGGGAA	Initial denaturation: 95℃ for 5 min; 32 cycles: 95℃ for 30s, 56℃ for 30s, 72℃ for 1min; final	1066 bp
(+)-β-caryophyllene synthase gene	MK861975	Forward: CAGATCACCTTA CCGGCGTT Reverse: TCGATCAGTTCC CTTTCCGC	Initial denaturation: 95℃ for 5 min; 32 cycles: 95℃ for 30s, 55℃ for 30s, 72℃ for 1 min;	975 bp
gyrB gene	MK861976	Forward: GAGGTCGTGCTG ACCGTGCTGCAC GCGGGCGGCAA GTTCGGC Reverse: GTTGATGTGCTG GCCGTCGACGTC GGCGTCCGCCAT	Initial denaturation: 95℃ for 5 min; 32 cycles: 95℃ for 30s, 65℃ for 30s, 72℃ for 1 min 30s; final extension: 72℃ for 10 min.	1284 bp

Supplementary Table S3. List of the oligonucleotide primers for qRT- PCR analysis in this study.

**Gene name**	**Gene functions**	**Primers used in the qRT-PCR**
aflR	Pathway regulator	F:AGCACTACAAACACTGACCCAC<br>R:CCAGCACCTTGAGAACGATAA
aflCa	Noranthrone monooxygenase, Norsolorinate-anthrone to norsolorinate	F: GCACCAATGGAGCCGTAT<br>R: GCGGTGTTCGTAGCGTTC
aflA	FAS alpha subunit, Acetate to polyketide	F: CGTGAGGTCAAGGCATTCCT<br>R: GACTTGGCCCCCCTTCTGT
aflS	Transcription enhancer, Pathway regulator	F: CCGAAGATTCCGCTTGGA<br>R: TGAAGACATGCAGCAAAAGGA
aflO	O-methyltransferase B, dihydrodemethylsterigmatocystin to dihydrosterigmatocystin	F: TGCTGTGGCATCCATTCAAA<br>R: GGACTGCGTCTTCCAAAAGG
aflD	NOR reductase, norsolorinic acid to averantin	F: ACTGCGACTCGGAAACTGATG<br>R: TGCTCCTCCCGCAATGTC
aflP	O-methyltransferase A, sterigmatocystin to O-methylsterigmatocystin	F: TGTGTCGAGTGATGTGGGACTAG<br>R: GCCACCCAGCTCAACCTACA
aflF	NOR dehydrogenase, norsolorinic acid to averantin	F: AAGATGCTGGGCACGTTTG<br>R: CATGGGTGAGGACGAATTGG
aflQ	Oxydoreductase, O-methylsterigmatocystin to AFB_1 and AFG1, dihydro-Omethylsterigmatocystin to AFB_2 and AFG_2	F: TTGCTGGGCTTGTGGATTC<br>R: GAGGAGGACGCGTGTCTTTG
aflX	Monooxygenase/oxidase, VA to DMST	F: ACCGCGTTGCACATCGT<br>R: TGGGTGTCCACAACCTTCGT
AccC	Acetyl-CoA carbosylase, Acetyl-CoA to Malonlyl-CoA	F: ATGGTAAGACCTGCCTGCTA<br>R: AGCGAGGATACCGAGGAT
β-tubulin	Endogenous control, Reference gene	F: AGCAGGCGAAGAAGGAGG<br>R: ACGCCACGCATTTGATCTTC

Reference: Gong, A. D., Wu, N. N., Kong, X. W., Zhang, Y. M., Hu, M. J., Gong, S. J., Dong. F. Y., Wang, J. H., Zhao, Z. Y., and Liao, Y. C. (2019) Inhibitory effect of volatiles emitted from *Alcaligenes faecalis* N1-4 on *Aspergillus flavus* and aflatoxins in storage. *Front. Microbiol.* 10:1419. doi: 10.3389/fmicb.2019.01419

Supplementary Table S4. Effects of the volatile organic compounds (VOCs) from the autoclaved wheat grains (AWG) cultures of *Streptomyces yanglinensis* 3-10 and soil amendment with the AWG cultures of this strain on peanut seedlings growth.

Character	Fumigation with the VOCs			Soil amendment with live culture of *S. yanglinensis* 3-10	
	AWG culture of *S. yanglinensis* 3-10 at 12 g/L *n* =16	AWG culture of *S. yanglinensis* 3-10 at 34 g/L *n* =16	Fresh AWG *n* =16	AWG culture of *S. yanglinensis* 3-10 at 5% (w/w) *n* =16	Fresh AWG *n* =16
Height (cm)	14.1 ± 1.9	11.8 ± 2.2*	14.9 ± 1.70	16.1 ± 4.6	16.7 ± 3.2
TDW (g per seedling)	0.39 ± 0.09	0.38 ± 0.10	0.41 ± 0.11	1.02 ± 0.25	1.05 ± 0.26

*Significantly different ($P < 0.05$) in comparison with that in the control treatment according to Student's *T* test. TDW =Total dry weight

II. Supplementary Figures

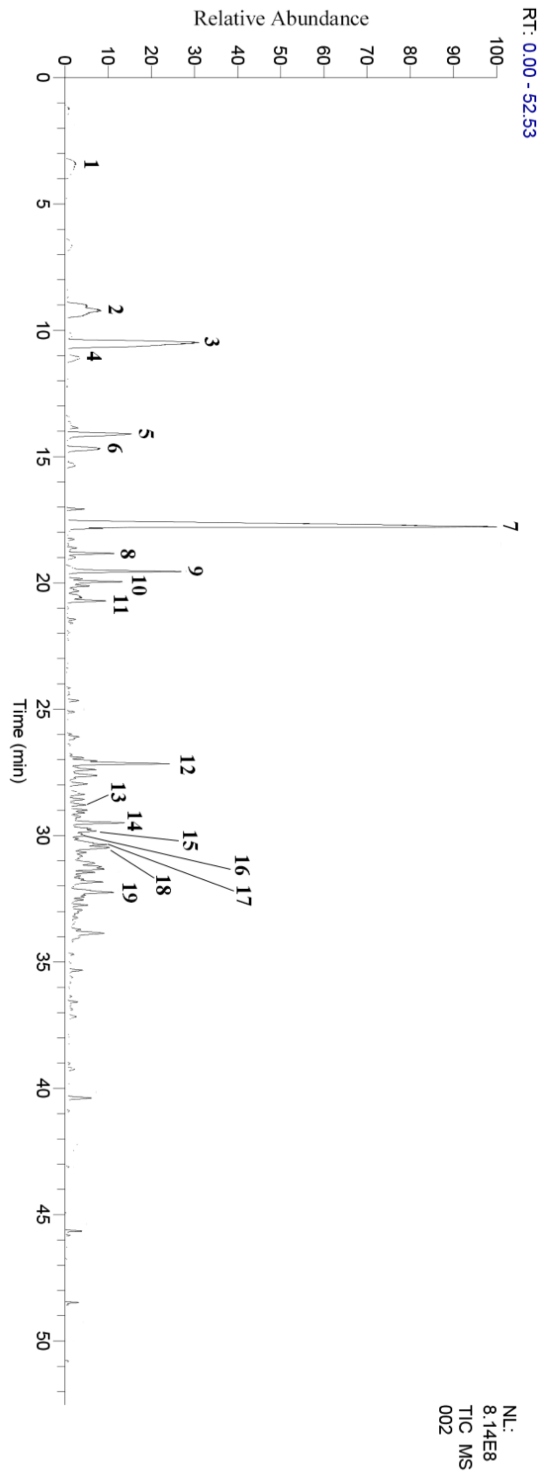

Supplementary Figure S1. A SPME-GC-MS chromatogram showing 19 volatile organic compounds (VOCs) emitted from a 10-day-old AWG culture of *Streptomyces yanglinensis* 3-10 (AWG = autoclaved wheat grains). Chemical identity of the labeled VOCs was listed in Table 1. **Peak 1**, methyl 2-methylbutyrate (M2M); **Peak 3** = 2-methyl-2-bornene (2-M2B); **Peak 6**, 2-Phenylethanol (2-PE); **Peak 7**, 2-methylisoborneol (2-MIB); **Peak 12**, *trans*-1,10-dimethyl-*trans*-9-decalinol (geosmin); **Peak 13**, (+)-β-caryophyllene

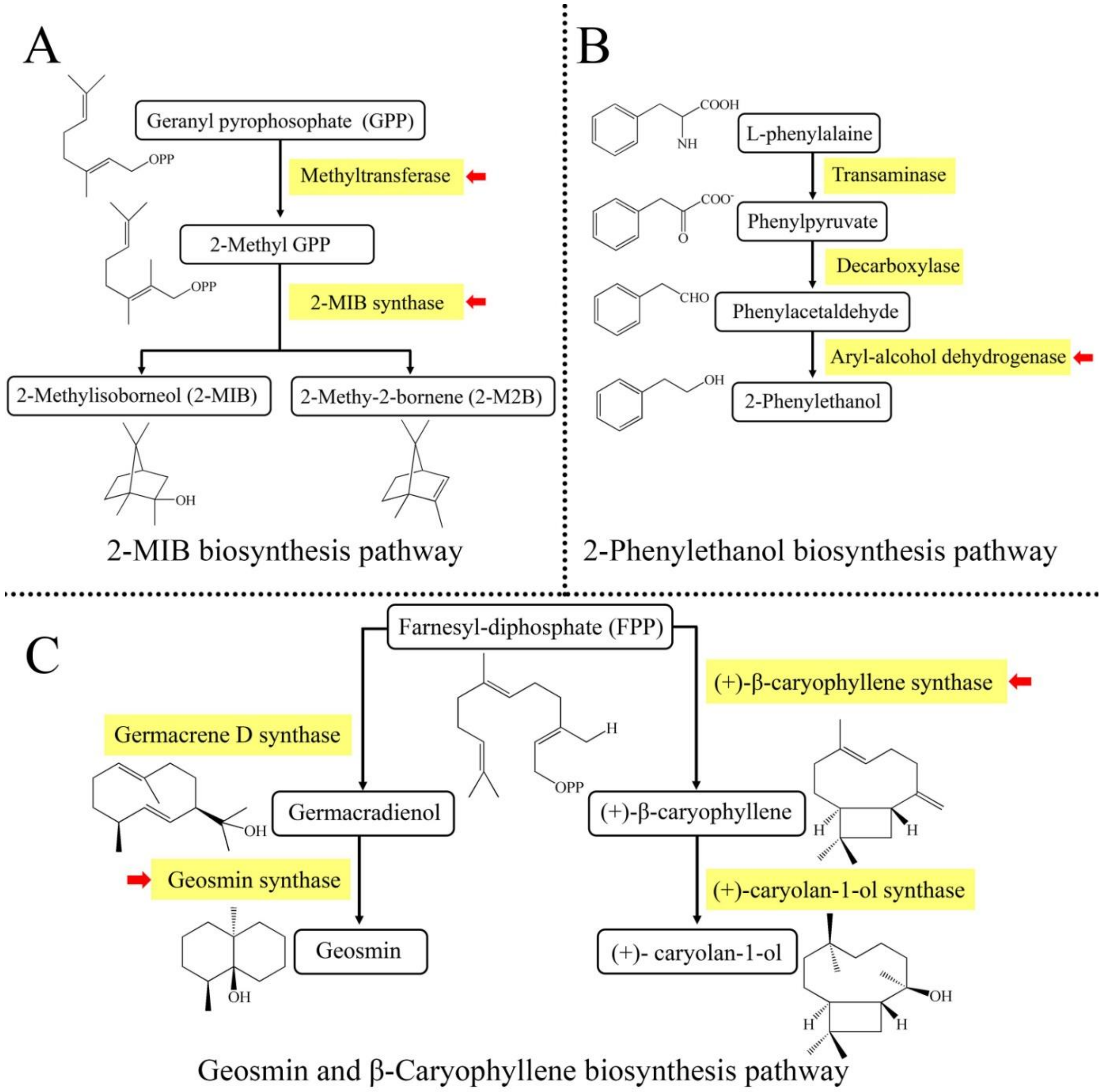

Supplementary Figure S2. Biosynthesis pathways of selected VOCs from *S. yanglinensis* 3-10. The red arrows indicate the genes used for detection of expression by RT-PCR. References for the biosynthesis pathways: **A**, from Komatsu et al. 2008. Proceedings of the National Academy of Sciences of the United States of America, 105: 7422-7427; **B**: from Zhang et al. 2014, BMC Biotechnology 14: 30; **C**, from Jiang et al. 2007. Nature Chemical Biology 3: 711-715; and Nakano et al. 2011. The Journal of Biological Chemistry 286: 27980-27987.

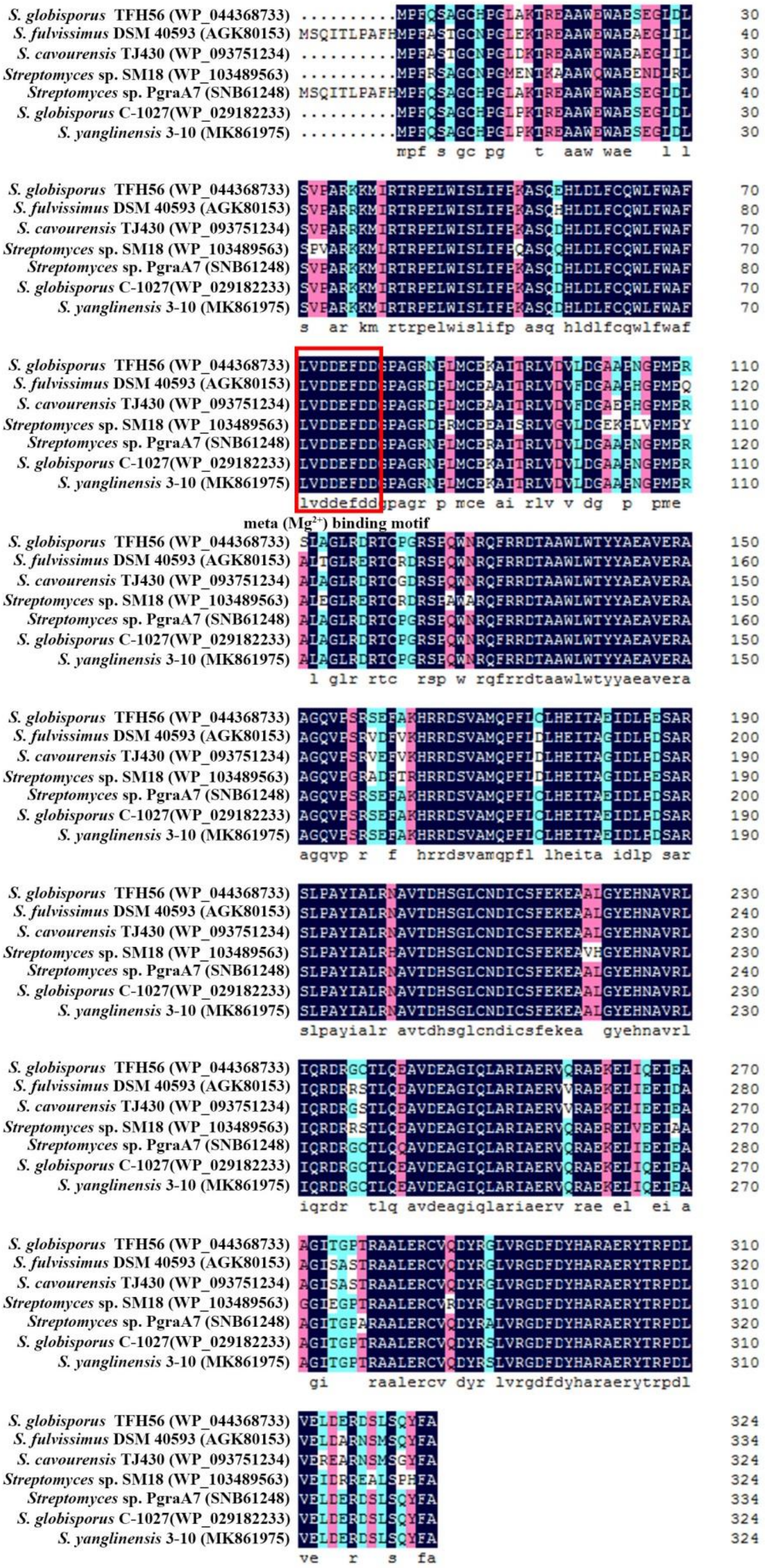

Supplementary Figure S3. (+)-beta-Caryophyllene synthase AA sequence alignment

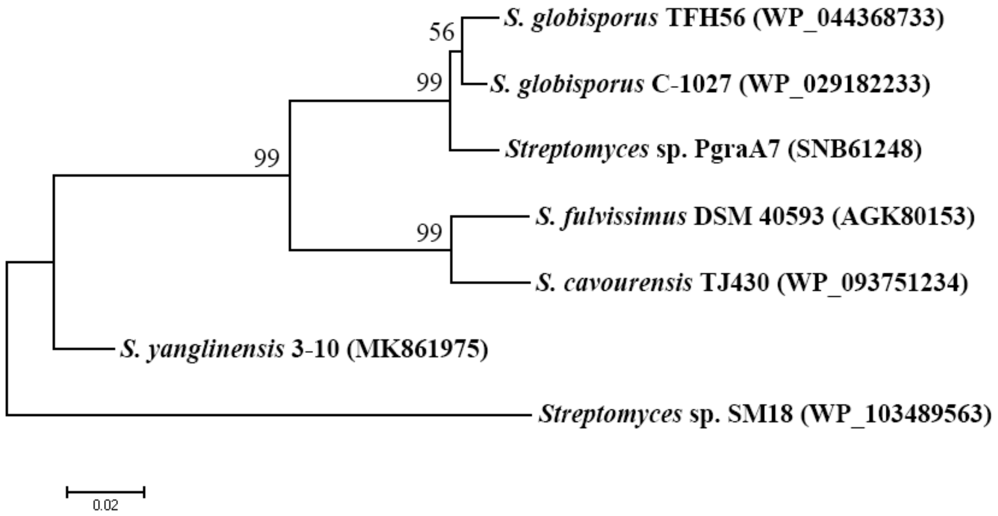

Supplementary Figure S4. A phylogenetic tree based on AA sequence of (+)-beta-caryophyllene synthase using the maximum likelihood method. The numbers at the nodes in that tree indicate the bootstrap percentages (*n* = 1, 000). The bar indicates the average nucleotide change per site.

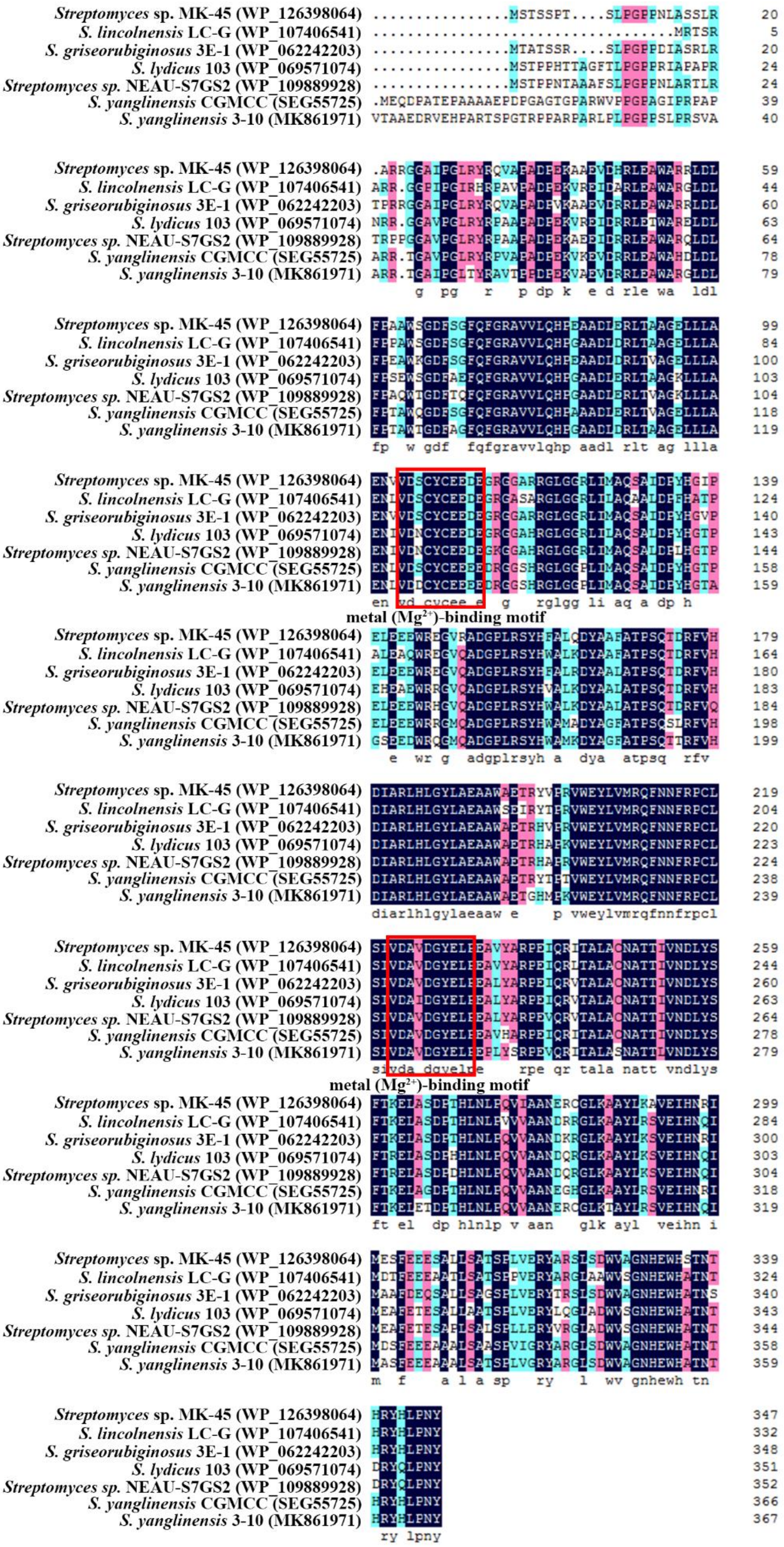

Supplementary Figure S5. Alignment of the amino acid sequences of 2-MIB synthase in *Streptomyces* species

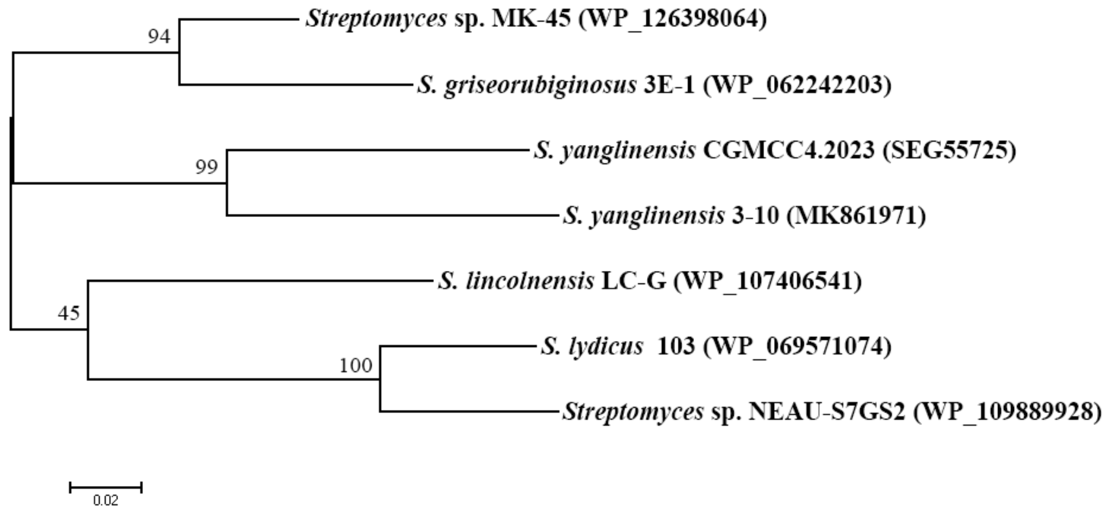

Supplementary Figure S6. A phylogenetic tree inferred based on the amino acid sequences of 2-MIB synthase in *Streptomyces* using the maximum likelihood method. The numbers at the nodes in that tree indicate the bootstrap percentages (*n* = 1, 000). The bar indicates the average nucleotide change per site.

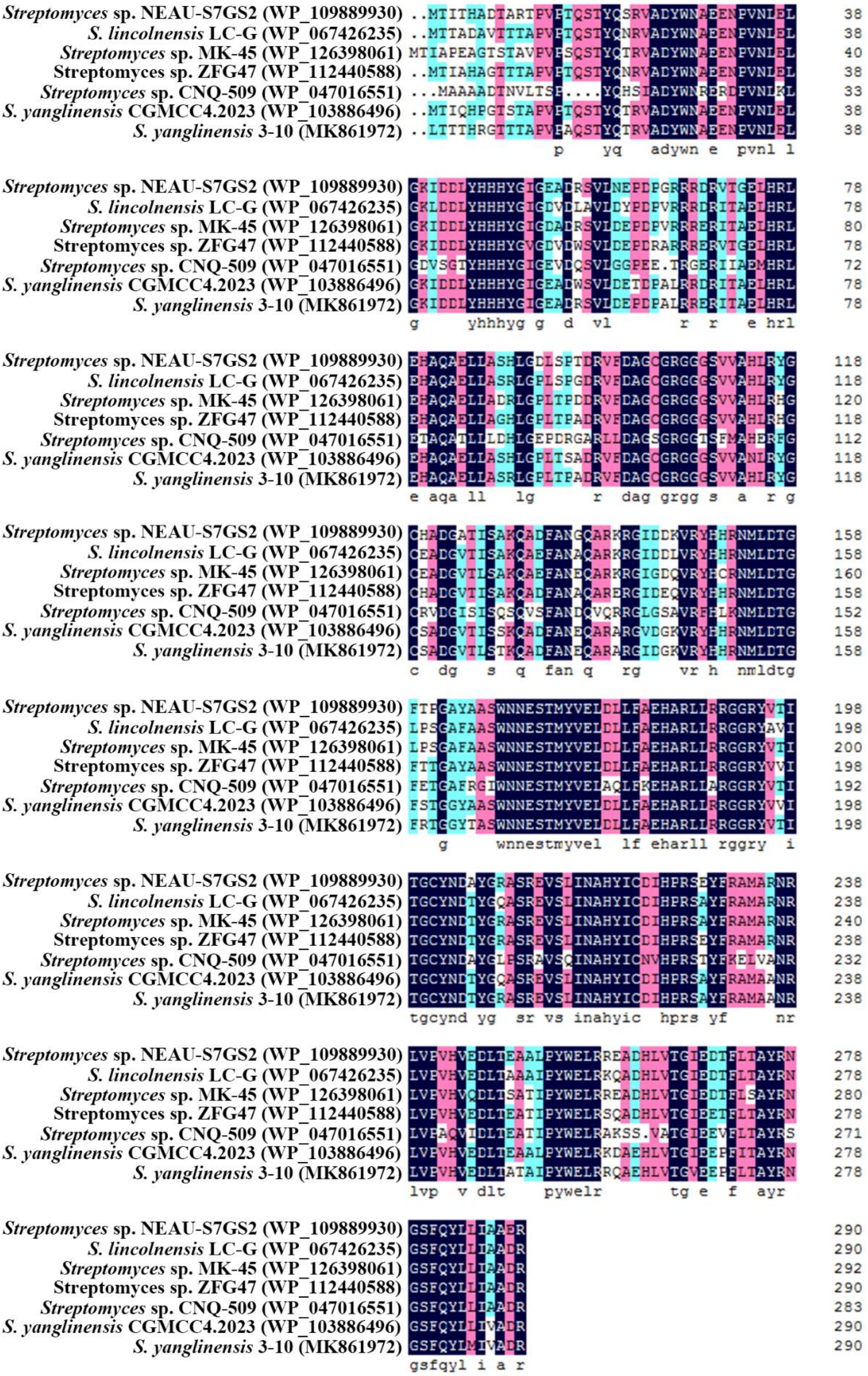

Supplementary Figure S7. Methyltransferase AA sequence alignment

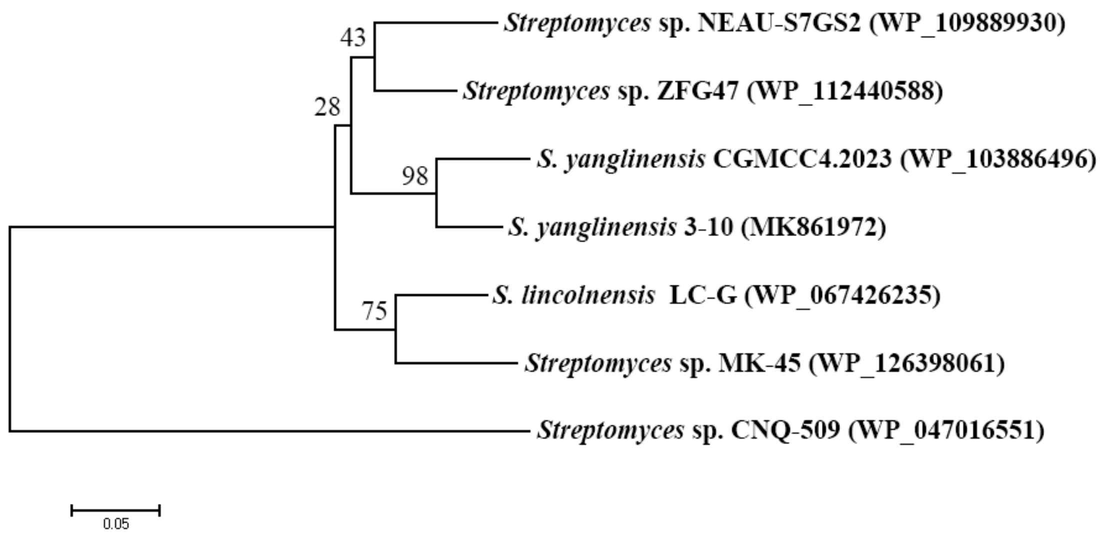

Supplementary Figure S8. A phylogenetic tree based on AA sequence of methyltransferase using the maximum likelihood method. The numbers at the nodes in that tree indicate the bootstrap percentages (n = 1, 000). The bar indicates the average nucleotide change per site.

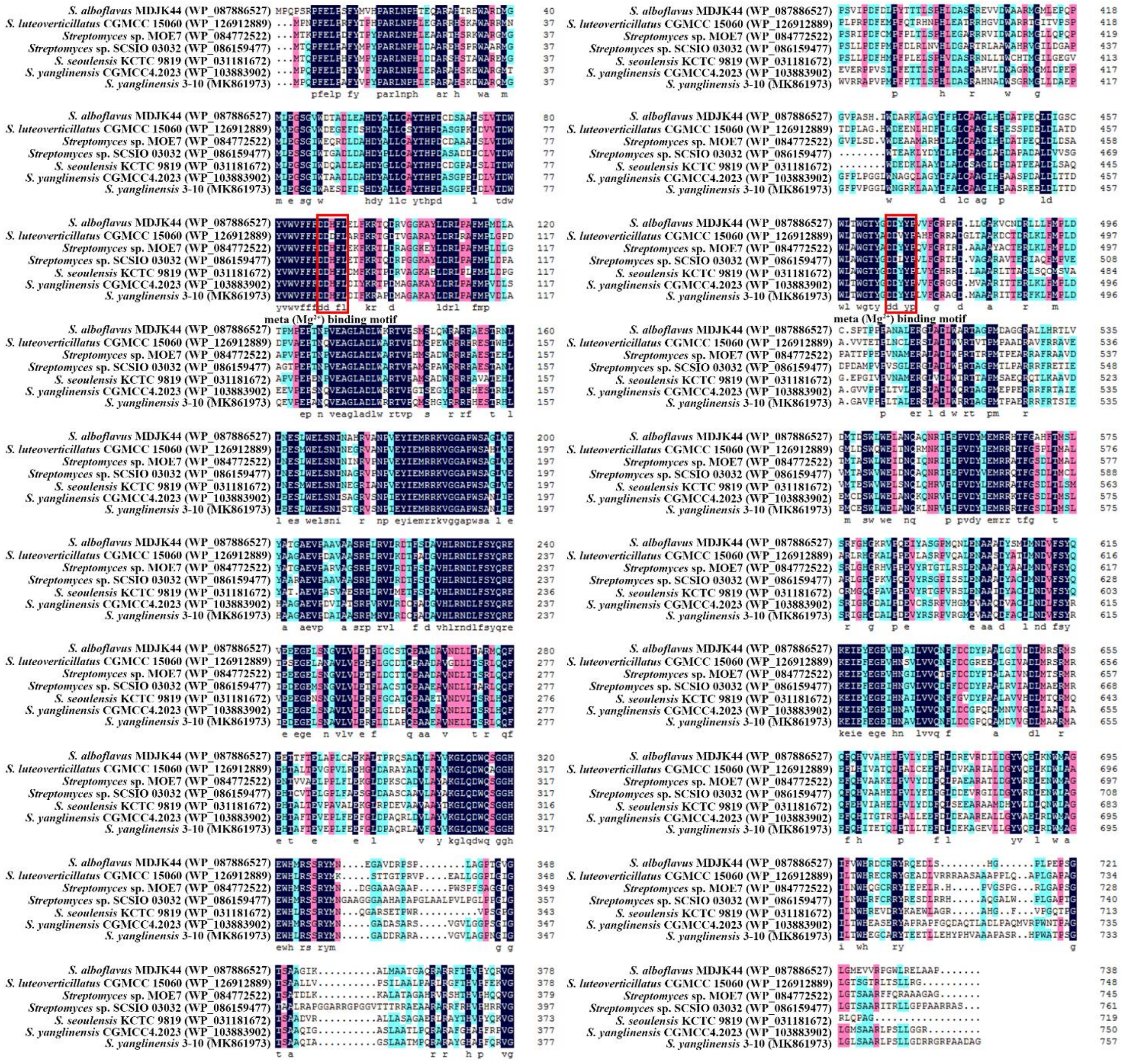

Supplementary Figure S9. Geosmin synthase AA sequence alignment

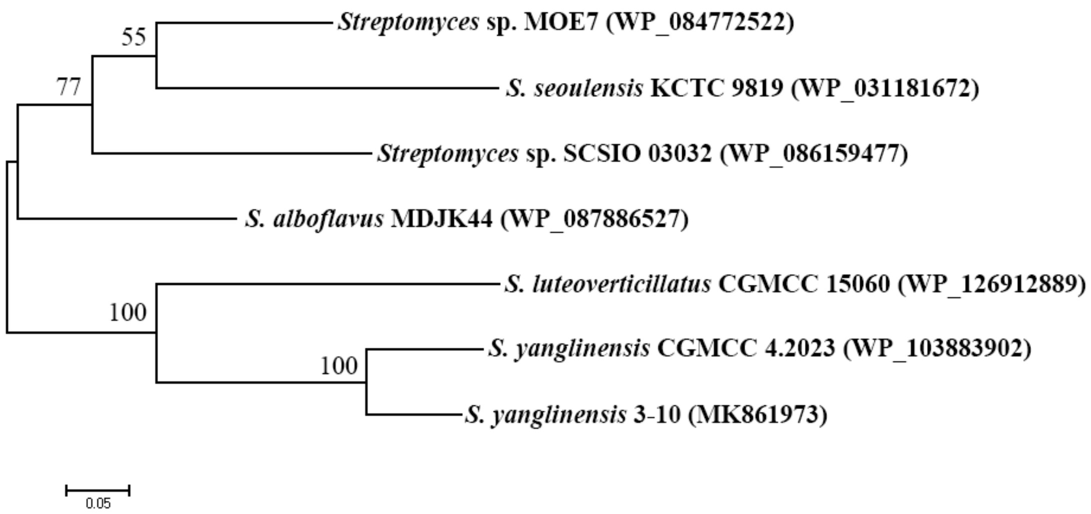

Supplementary Figure S10. A phylogenetic tree based on AA sequence of geosmin synthase using the maximum likelihood method. The numbers at the nodes in that tree indicate the bootstrap percentages (*n* = 1, 000). The bar indicates the average nucleotide change per site.

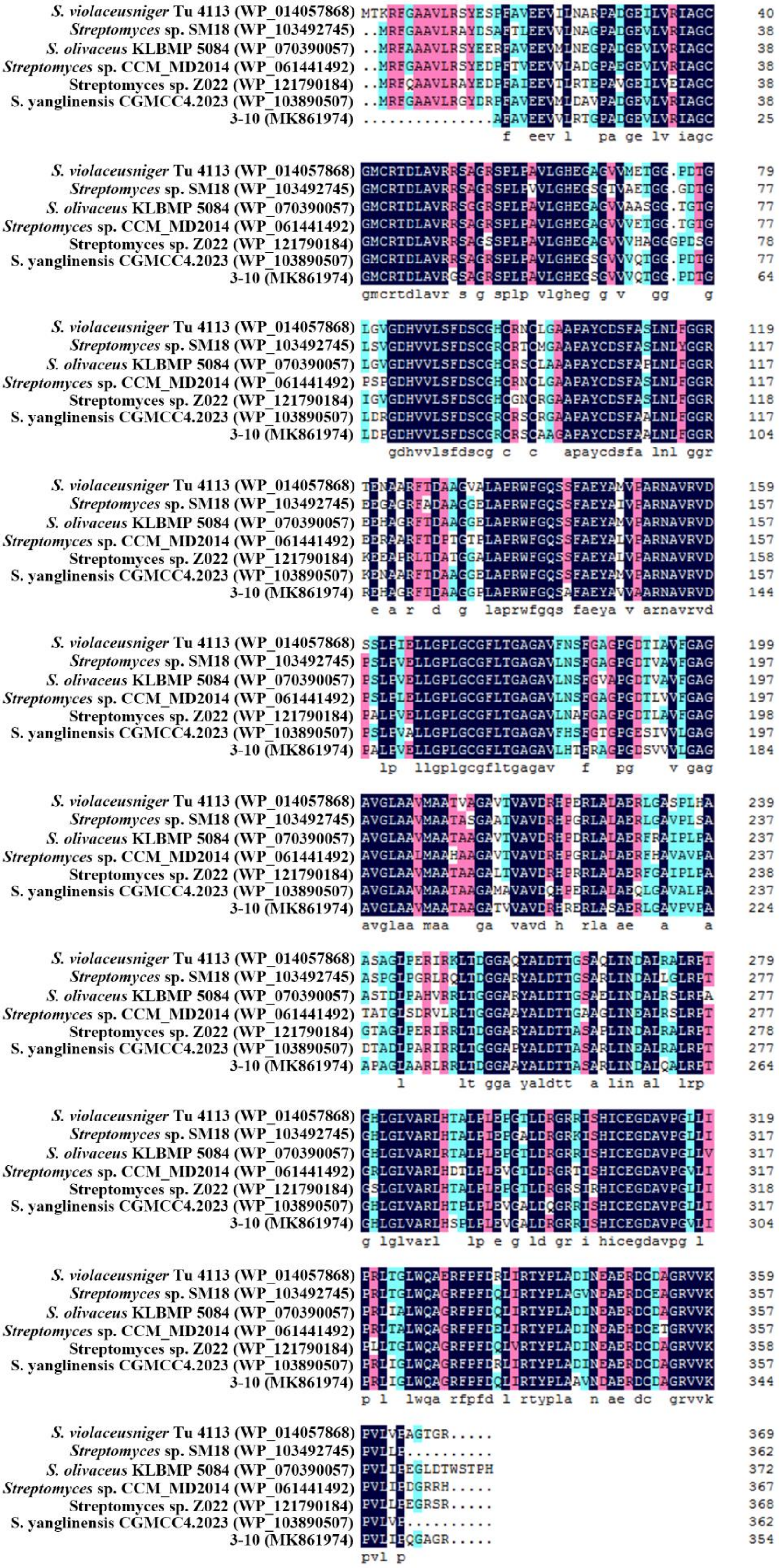

Supplementary Figure S11. Aryl-alcohol dehydrogenase (2-phenylethanol synthase) AA sequence alignment

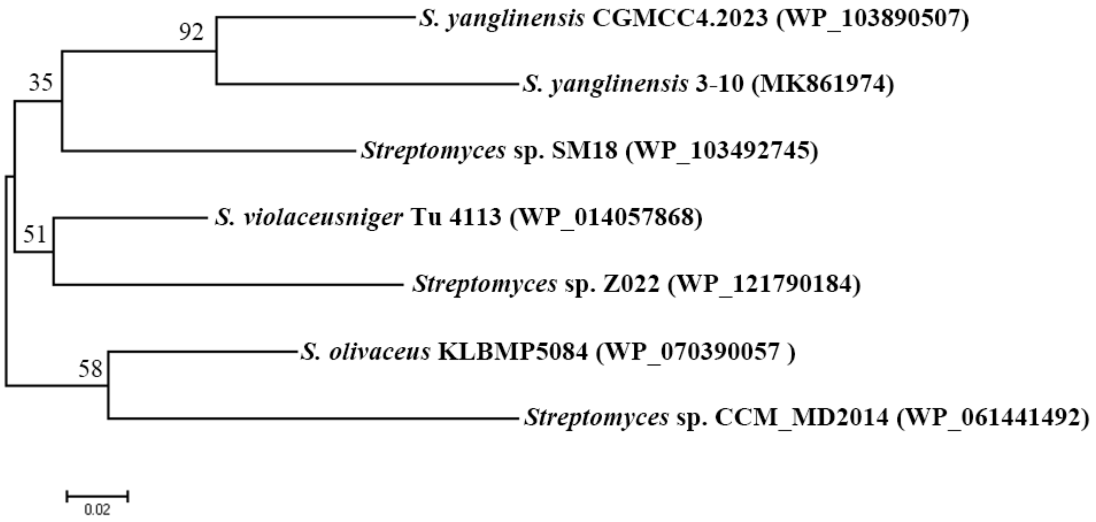

Supplementary Figure S12. A phylogenetic tree based on AA sequence of Aryl-alcohol dehydrogenase (2-phenylethanol synthase) using the maximum likelihood method. The numbers at the nodes in that tree indicate the bootstrap percentages (*n* = 1, 000). The bar indicates the average nucleotide change per site.

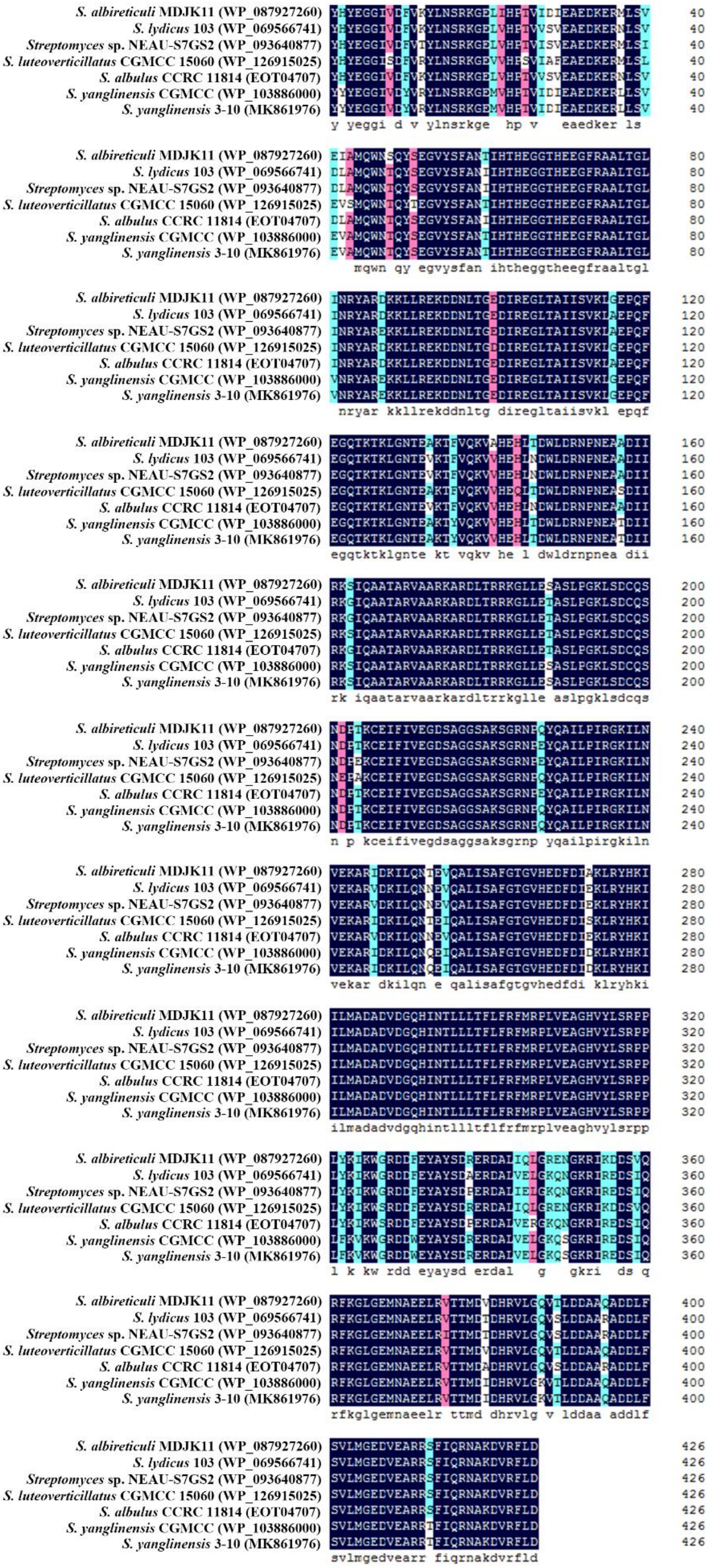

Supplementary Figure S13. gyrB subunit AA sequence alignment

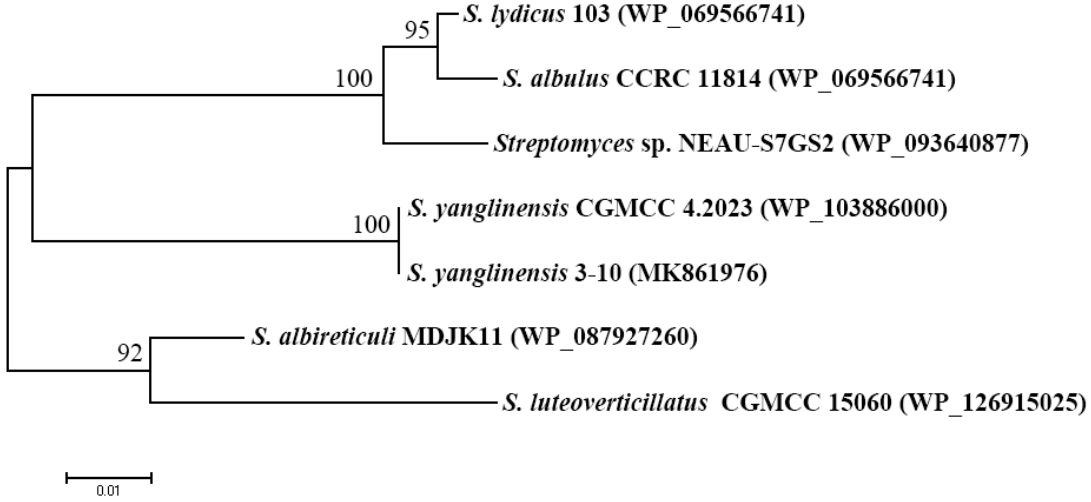

Supplementary Figure S14. A phylogenetic tree based on AA sequence of gyrB subunit using the maximum likelihood method. The numbers at the nodes in that tree indicate the bootstrap percentages (*n* = 1, 000). The bar indicates the average nucleotide change per site.

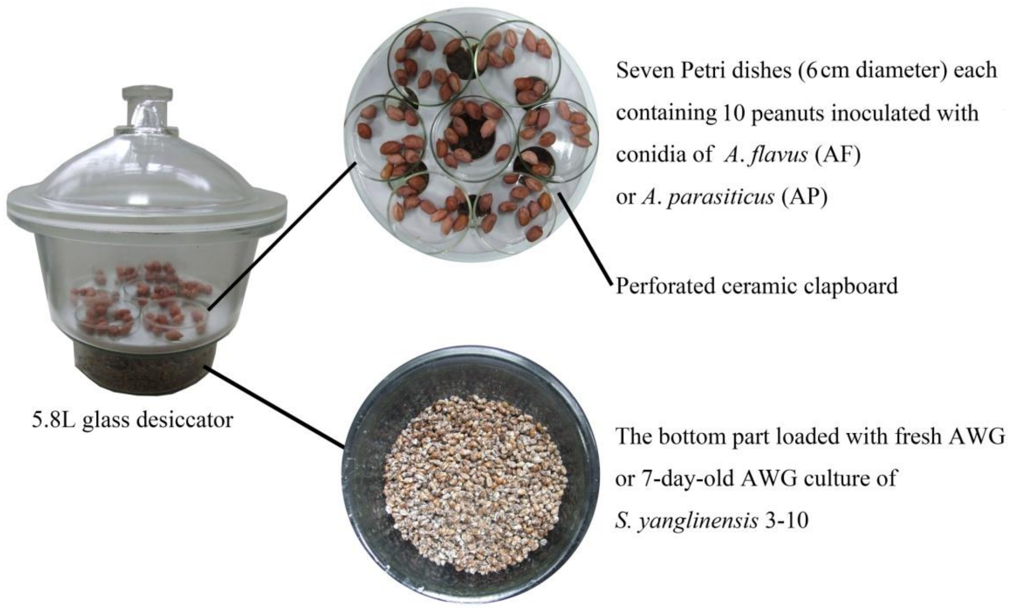

Supplementary Figure S15. A glass desiccator showing set-up of the bioassay for determination of the efficacy of the volatiles from *Strptomyces yanglinensis* 3-10 in suppression of colonization of peanut kernels by *Aspergillus flavus* and *A. parasiticus*.

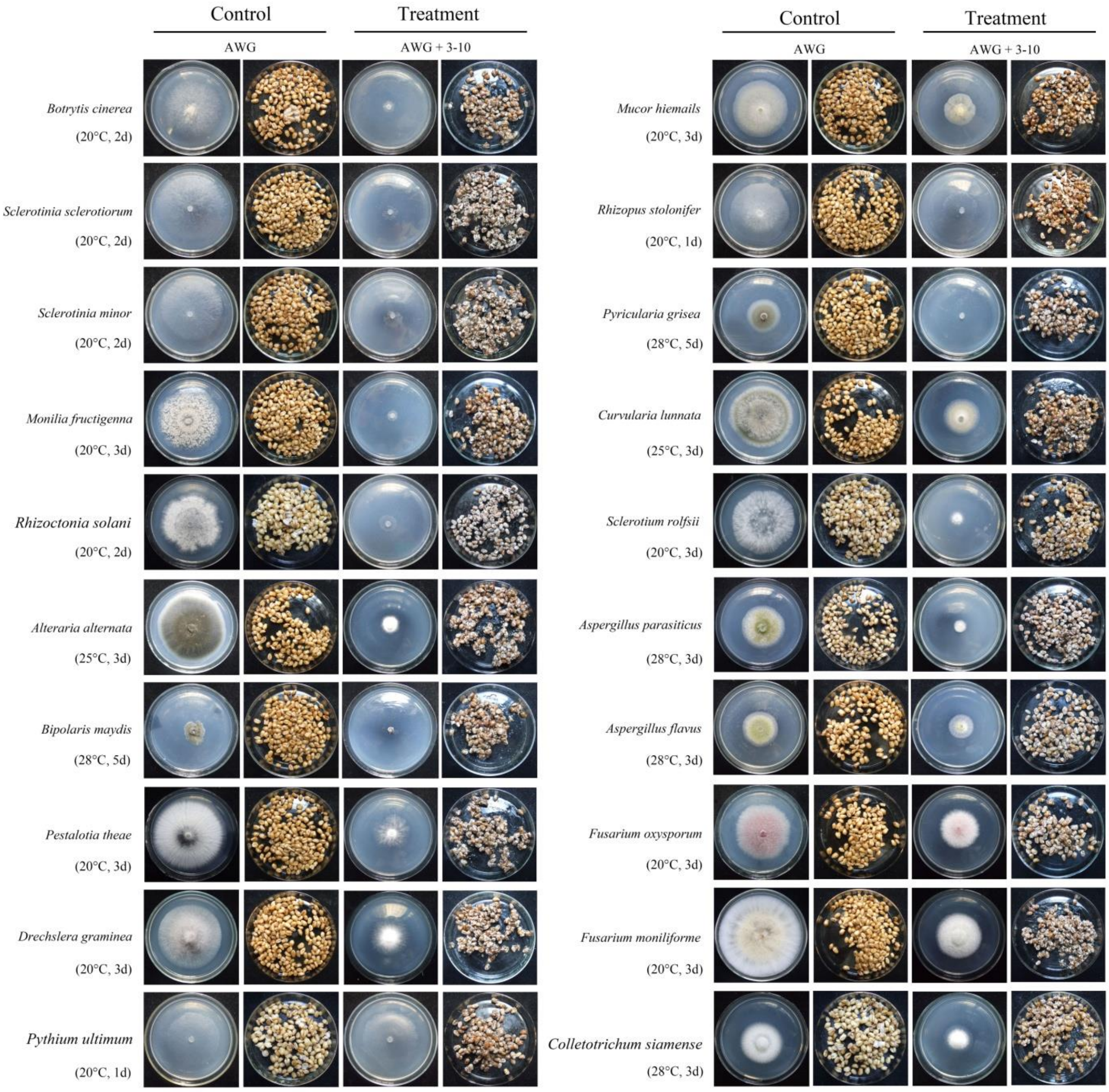

Supplementary Figure S16. Antifungal spectrum of the VOCs from *Streptomyces yanglinensis* 3-10.

Control = fresh AWG, Treatment = fumigated with the VOCs from the AWG cultures of *S. yanglinensis* 3-10

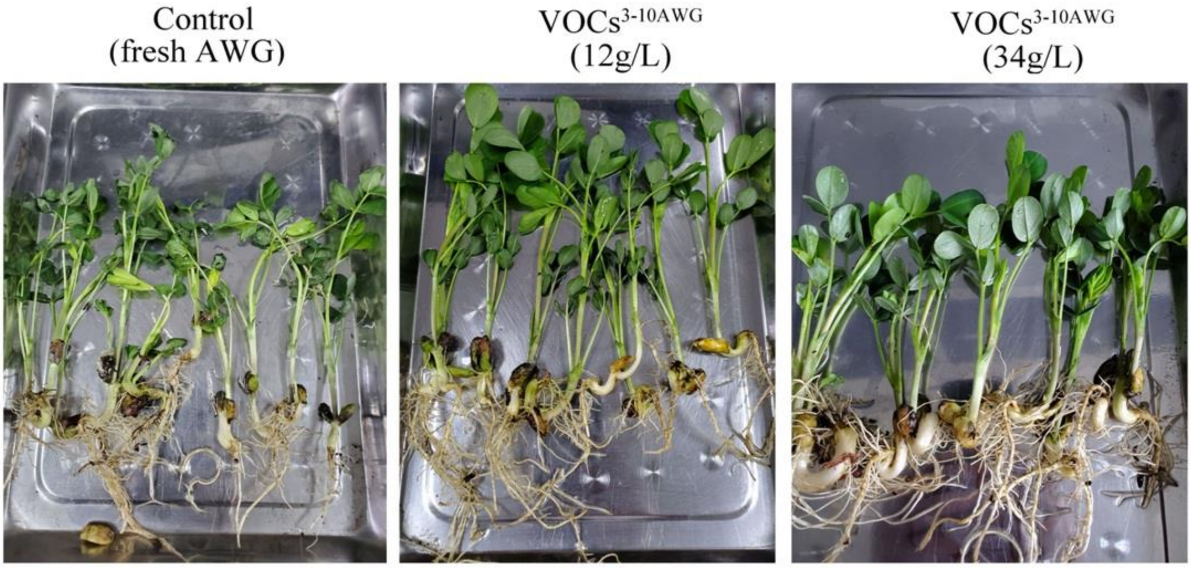

Supplementary Figure S17. Effect of the VOCs from the 7-day-old AWG culture of *Streptomyces yanglinensis* 3-10 ($VOCs^{3\text{-}10AWG}$). AWG = autoclaved wheat grains. The seedlings were fumigated for seven days under the VOCs from fresh AWG or the AWG culture of *Streptomyces yanglinensis* 3-10.
